# Polyploid cell dynamics and death before and after PEG-treatment of a NIH/3T3 derived culture: vinblastine effects on the regulation of cell subpopulations heterogeneity

**DOI:** 10.1186/s13008-023-00100-y

**Published:** 2023-10-30

**Authors:** Alessandra Spano, Luigi Sciola

**Affiliations:** Present Address: Department of Biomedical Sciences, Sassari - University of Sassari, Via Muroni 25, 07100 Sassari, Italy

**Keywords:** Polyploidy, Cell fusion, Mitotic anomalies, Autophagic-like death

## Abstract

**Background:**

Neoplastic subpopulations can include polyploid cells that can be involved in tumor evolution and recurrence. Their origin can be traced back to the tumor microenvironment or chemotherapeutic treatment, which can alter cell division or favor cell fusion, generating multinucleated cells. Their progeny, frequently genetically unstable, can result in new aggressive and more resistant to chemotherapy subpopulations. In our work, we used NIHs cells, previously derived from the NIH/3T3 line after serum deprivation, that induced a polyploidization increase with the appearance of cells with DNA content ranging from 4 to 24c. This study aimed to analyze the cellular dynamics of NIHs culture subpopulations before and after treatment with the fusogenic agent polyethylene glycol (PEG), which allowed us to obtain new giant polyploid cells. Successively, PEG-untreated and PEG-treated cultures were incubated with the antimicrotubular poison vinblastine. The dynamics of appearance, decrease and loss of cell subpopulations were evaluated by correlating cell DNA content to mono-multinuclearity resulting from cell fusion and division process alteration and to the peculiarities of cell death events.

**Results:**

DNA microfluorimetry and morphological techniques (phase contrast, fluorescence and TEM microscopies) indicated that PEG treatment induced a 4–24c cell increase and the appearance of new giant elements (64–140c DNA content). Ultrastructural analysis and autophagosomal–lysosomal compartment fluorochromization, which allowed us to correlate cytoplasmic changes to death events, indicated that cell depletion occurred through distinct mechanisms: apoptotic death involved 2c, 4c and 8c cells, while autophagic-like death involved intermediate 12–24c cells, showing nuclear (lobulation/micronucleation) and autophagic cytoplasm alterations. Death, spontaneously occurring, especially in intermediate-sized cells, was increased after vinblastine treatment. No evident cell loss by death events was detected in the 64–140c range.

**Conclusions:**

PEG-treated NIHs cultures can represent a model of heterogeneous subpopulations originating from cell fusion and division process anomalies. Altogether, our results suggest that the different cell dynamics of NIHs subpopulations can affect the variability of responses to stimuli able to induce cell degeneration and death. Apoptptic, autophagic or hybrid forms of cell death can also depend on the DNA content and ability to progress through the cell cycle, which may influence the persistence and fate of polyploid cell descendants, also concerning chemotherapeutic agent action.

## Background

Multicellular organisms tend to maintain an appropriate cell size that can show general uniformity within most animal tissues. Mammalian cells can regulate their size through a system connected to the succession of the cell cycle phases and, therefore, to the division process that requires a preventive increase in cell mass. In general, changes in cell size and nuclear DNA content affect several aspects of the structure and function of the cells. Homeostasis of cell size in different tissues involves different signaling pathways that can contribute to the regulation of proliferative activity: larger cells grow less than small ones. The increase in cell size, therefore, affects signaling pathways that regulate the expression of genes related to cell cycle progression. Experimental evidence shows that cell size regulation takes place around the G1-S transition of the cell cycle [1]. However, the different biological implications of adequate cellular size in tissues are not fully understood [2, 3].

Regarding neoplastic cell populations, several studies have shown that cells constituting tumors exhibit evident differences in both the size and characteristics of the genome. They are complex biological systems in which cancer cells with different sizes and DNA contents are present. The relative cellular subpopulations are heterogeneous and consist of stem cells, polyploids, and bulk cells. The latter is usually the prevalent component within solid tumor cell populations and frequently shows a high degree of aneuploidy, with an increase in chromosome number.

Cancer stem cells (CSCs) play a decisive role in maintaining the cellular heterogeneity and malignancy of various tumors, fueling growth and resistance to radiotherapy and chemotherapy treatment [4]. In addition to CSCs, this property has been attributed to a component defined as ‘‘stemloids’’ consisting of proliferating cells originating from progenitors that were found to be able to reactivate the self-renewal capability. In these cells, a transient polyploidy mechanism capable of originating the clonogenic growth of neoplastic cells after genotoxic treatment has been documented by some authors. Other studies still conducted on lymphoma cell lines have investigated the role of pluripotency and self-renewal of stem genes OCT4, SOX2, and NANOG in this polyploidy-dependent survival mechanism. The results of the study indicated that polyploid cells are refractory to apoptosis and able to overcome senescence by implementing different mechanisms of mitotic division and transmitting the upregulated self-renewal stem genes OCT4, SOX2, and NANOG to their descendants [5–7].

 Atypical polyploid multinucleated fibroblasts have been described in several previous works, referring to mammary fibroepithelial neoplasms [8], to the stroma of benign breast tumors [9], to connective tissue diseases due to fibrotic phenomena or in the senescence of in vitro cell models [10].

Regarding in vitro situations, some studies have indicated that multinuclearity is reached by cell fusion or due to alteration of cytokinetic processes depending on the cell culture conditions. It has already been demonstrated how the subtraction of serum from the culture medium can lead to polyploidy induction [11]. Alternatively, giant cell formation has been described in cultures of senescent fibroblasts as a consequence of alterations in cell division processes, with mitoses not accompanied by cytokinesis [12]. Then, polyploid giant cells can derive from subsequent cycles of DNA replication with cytokinesis failure and can be associated with mitotic checkpoint defects, as occurs in cancer cells, in which an increase in their chromosome number can be observed [13]. At present, it is not yet clear whether these cell features can cause the onset of tumors or whether neoplastic transformation determines the origin of giant polyploid/aneuploid cells [14, 15]. In this context, it has been speculated that cell fusion may also play a role in the transition from polyploidy to aneuploidy and hence in tumor progression [16]. Therefore, this pathological process appears to be related to the increase in genome instability detected in polyploid and aneuploid cells, which, through altered division processes, can originate giant cells with a significant contribution to the cellular heterogeneity that characterizes some tumors. Cells with high levels of ploidy show a substantial inability to undergo mitotic processes, which, when not correctly performed, can lead to mitotic catastrophe and therefore to cell death [17]. Although polyploidy and aneuploidy can generally represent significant prognostic factors for the evolution of different tumor types, the biological role of neoplastic cells in these conditions has yet to be fully elucidated. Therefore, there is a clear need to expand knowledge on the role of polyploid/aneuploid cells in the origin and chemoresistance of some neoplasms. Some reports have indicated that some polyploidy/aneuploid cells that appear to be resistant to chemotherapy can proliferate and lead to the reappearance of tumors. On the other hand, drugs capable of affecting DNA structure and mitotic dynamics can induce the formation of polyploidy/aneuploid cells [18] that can constitute subpopulations with aggressive characteristics and play an important role in tumor negative evolution. Then, the origin of these cells can have multiple causes related to the tumor microenvironment or to chemotherapy itself [19].

Starting from these assumptions, interest in deepening the biological aspects underlying the development of polyploid/aneuploid giant cells and in analyzing cell death pathways, also related to antiproliferative and apoptogenic agent use, emerges. Further knowledge in this area could help to consolidate a new approach for cancer therapy for some types of tumors with very heterogeneous cancer cell subpopulations. In some tumors, such as those of mesenchymal origin, the highly variable response to chemotherapy could depend on the different drug sensitivities of the various cancer cell subsets.

In our laboratory, we have been working for some time in this area, and in recent years, we have used a culture derived from the NIH/3T3 mouse fibroblast line, here named NIHs, obtained after prolonged serum deprivation as an experimental model [20].

The withdrawal of serum caused massive cell detachment and death of almost all cells constituting the culture. When serum was restored, the surviving cells (NIHs) re-entered the cell cycle and recovered their proliferative activity. In comparison with the original NIH 3T3 cell line, in the successive stages of propagation of the NIHs culture, the appearance of polyploid cells with DNA content values ranging from 4 to 24c [20] was detected. Some elements have shown the characteristics of cellular senescence; however, the greater fraction of NIHs cells has shown a lower adhesion capacity toward the growth substrate (compared to the original NIH 3T3 line) and the tendency to form foci of actively proliferating fibroblasts. Cells isolated from these areas can grow in suspension and give rise to aggregates in the form of spheroids. For this set of reasons, it seemed interesting to use this cell line even if it is not made up of tumor cells.

Furthermore, given our interest in the biology of polyploid cells and since their fraction is usually relatively low in NIHs cultures, the first objective of the present work was to try to increase their number. For this purpose, we evaluated the effects of the fusogenic agent PEG.

The chemical methods based on PEG-mediated cell fusion are, in practice, alternatives to those with a biological basis, in which fusogen viruses are used [21], or those with a physical basis, involving instead the use of electric currents applied to the cells. Unlike PEG, which acts as a dehydrating agent that allows close contact of membranes, leading to the formation of small cytoplasmic bridges between cells, electrofusion uses pulses of alternating and direct currents or localized electric fields to merge plasma membranes through electroporation [22]. Each method has its advantages and disadvantages. PEG-mediated cell fusion is a simple and efficient technology and does not require expensive instrumentation. This approach, which can be performed on adherent and suspension cells [23], has been used widely for obtaining somatic cell hybrids, antibody production [24, 25] nuclear transfer in mammalian cloning [26], and the production of osteoclasts in vitro [27]. However, the procedure requires conducting tests aimed at balancing the fusion efficiency with the minimum level of cytotoxicity (with a negligible level of cytotoxicity) possibly induced by PEG, depending on the different cellular characteristics.

In our work, in addition to these tests, a series of preliminary experiments were aimed at establishing the most suitable protocol treatment to increase polyploid giant cells.

After this phase, we chose the experimental condition in which the microfluorimetric measurements of NIHs polyploid cells also showed a DNA content ranging from 4 to 140c (reference condition). The persistence of different polyploid cell subpopulations was evaluated during PEG-treated culture progression after cycles of cell propagation by trypsinization. The second objective of this study was to characterize the dynamics of the appearance, decrease and loss of the different cellular subpopulations of NIHs cells, also considering the possible origin mechanisms of polyploid cells and their ability to produce progeny. In the evaluation of these aspects, the DNA content was correlated to the conditions of mono- and multinuclearity and to the peculiarities of the cell death events.

The analysis of the general cell morphology was performed by phase-contrast and fluorescence microscopy, while some details of the structure of the nucleus and cytoplasm were examined by transmission electron microscopy (TEM). The functionality of the lysosomal compartment and the detection of autophagy were determined under conditions of cell viability using acridine orange.

In subsequent experiments, untreated and PEG-treated (in the reference condition) NIHs cultures were subjected to incubation with VBL, a cytostatic agent capable of inducing perturbations on the microtubular network with influences not only on the mitotic activity but also on the cellular structure itself. By considering the significant cellular heterogeneity of the PEG-treated NIHs culture in reference conditions, these experiments were aimed at testing the variability of the effects induced by VBL on the different cellular subpopulations. Tumor cell heterogeneity remains a fundamental obstacle that prevents the development of truly curative anticancer therapies [28].

## Results

### General morphological aspects and DNA content microfluorometry of NIHs cultures before and after PEG treatment

**Fig. 1:**
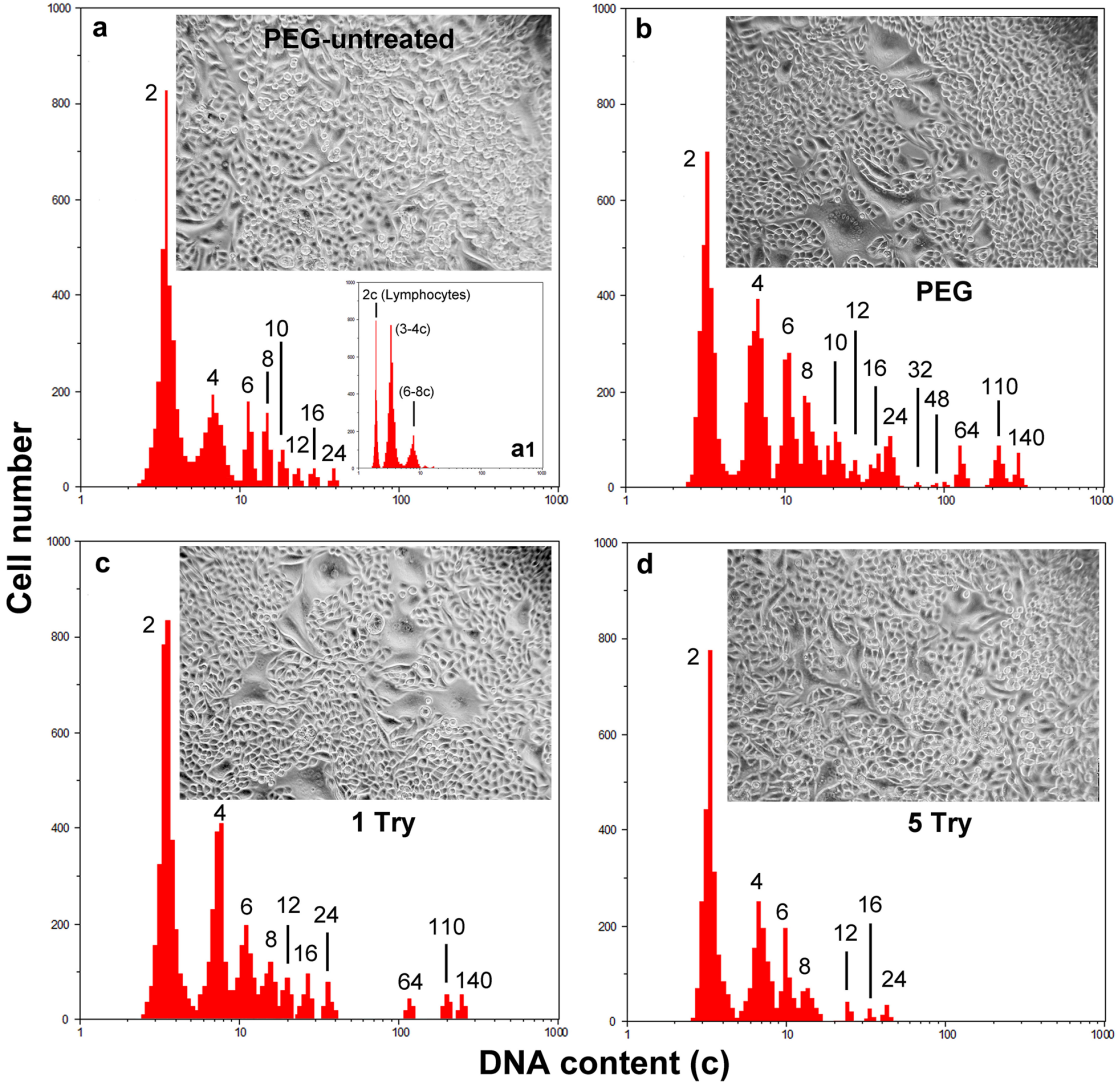
*Typical microfluorimetric analyses of the DNA cellular content after Hoechst 33342 fluorochromization.* In **a** PEG-untreated, in **b** PEG-treated NIHs cells, in **c** and **d** the measurements after one (**c)** and five (**d)** trypsinizations of PEG-treated cultures are reported, respectively. The smaller histogram (**a1**) inserted in **a** shows the microfluorimetric measurement of the NIH/3T3 cell line DNA content before serum starvation. During this analysis, a sample of mouse lymphocytes was measured in parallel to obtain the position of the value 2c in the abscissa axis (logarithmic scale). Although NIH/3T3 cells are hypertriploids (3–4c DNA content), NIHs cells derived from this line have been arbitrarily considered diploids to simplify the representation of data in various histograms. Therefore, in **a**, **b**, **c**, **d**, the first peak (3–4c DNA content) in the abscissa axis of microfluorimetric histogram was arbitrarily indicated as 2c. Overall histogram evaluations show the dynamics of the increase/decrease and/or appearance/disappearance of cells with different polyploid/aneuploid levels. Phase contrast micrographs inserted in **a**, **b**, **c**, and **d**, show the morphology of NIHs cultures under the respective experimental conditions

General morphological analysis of the NIHs cultures, obtained after serum starvation, was performed by phase contrast microscopy (micrographs in Fig. 1a, b, c, d). In the control condition (before PEG treatment), some dimensional heterogeneity of the cells was observed (micrograph in Fig. 1a).

The measurements of the DNA content performed on the original NIH/3T3 cell line before serum starvation (insert a1 in Fig. 1a and insert a2 in Fig. 9a1), allowed us to detect that these cells are hypertriploids with the first peak (G0/G1 phase) of DNA content histogram positioned in the 3–4c range. In the case of the NIHs line, given the presence of different cellular subpopulations, cells with 3–4c DNA content were arbitrarily considered diploids, with the first peak in the abscissa axis of the microfluorimetric histogram indicated as 2c. We considered this suitable to simplify the data representation of the different DNA content values of the polyploid NIHs cell subpopulations and to avoid having to represent ploidy values expressed by decimal numbers.

Taking into account this premise, in PEG-untreated NIHs cells, microflurometric measurements of DNA content showed the presence of polyploid elements with DNA content ranging from 4 to 24c (Fig. 1a).

**Fig. 2:**
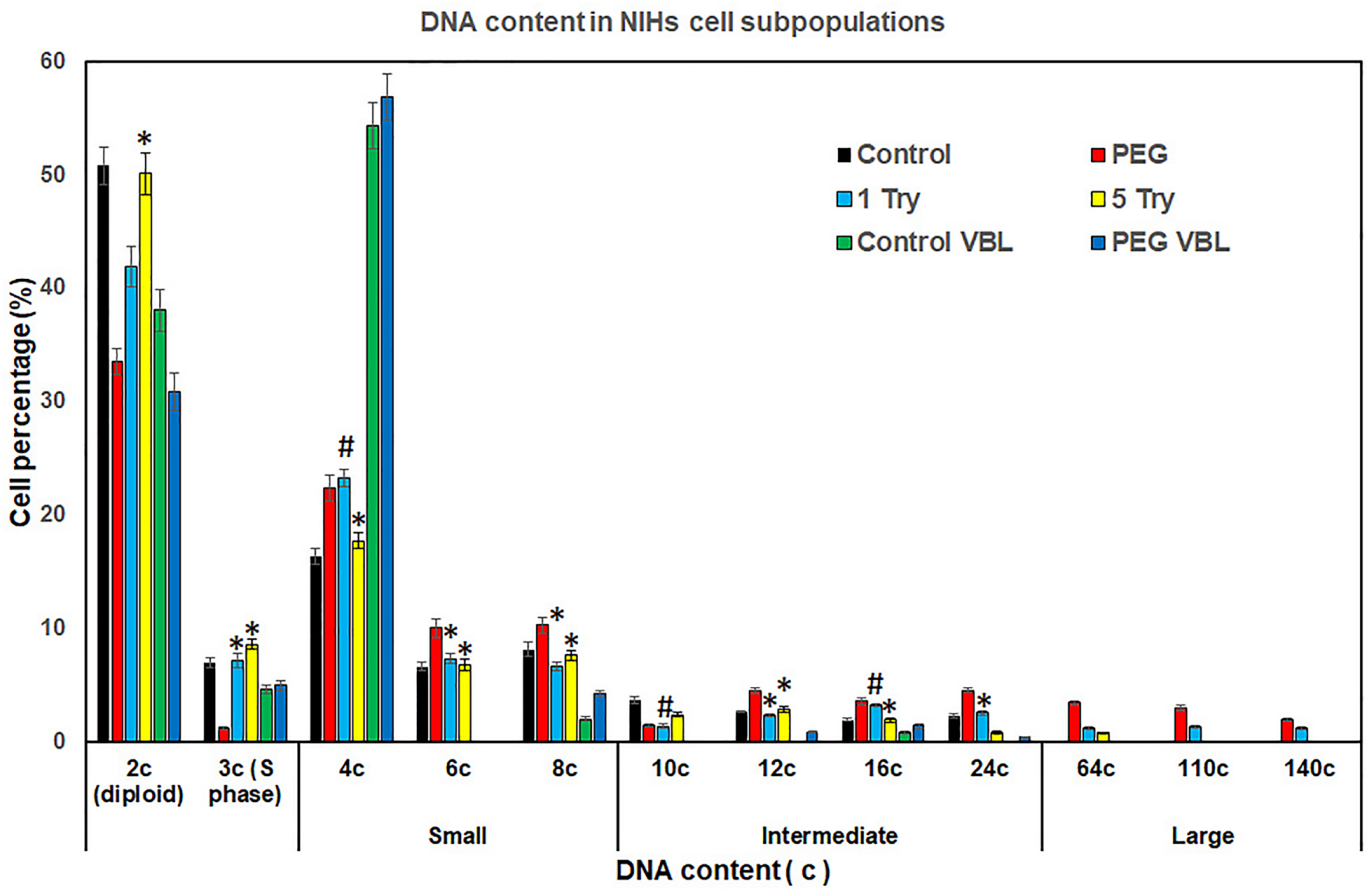
*DNA content in NIHs cell subpopulations.* Percentage of NIHs cells with different DNA content measured by microfluorimetry after Hoechst 33342 fluorochromization. The values, which refer to the main cell subpopulation of NIHs cultured in the different experimental conditions, represent the mean number of cells ± SEM of triplicate DNA content measurements. The asterisks (*) indicate the values that are not significantly different (P > 0.05 after Student's t-test) from the control (PEG-untreated cells). Hashtags (#) indicate the values of 1 Try, 5 Try, and PEG-VBL conditions that are not significantly different (P > 0.05 after Student's t-test) from PEG-treated cells (PEG)

The analysis 30 h after PEG treatment (Fig. 1b) showed that cell subsets included in the range 4c-24c underwent a general increase in their percentage incidence  (Fig. 2); PEG-induced cell fusion also resulted in the appearance of three new main giant cell subpopulations with 64c, 110c, and 140c DNA content (Fig. 1b; Fig. 2). Based on DNA content, polyploid elements were arbitrarily divided into small (4–8c), intermediate (10–24c), and large (64–140c) cells.

After PEG treatment, the percentage decrease of the 2c subpopulation may be in part the consequence of its involvement in fusion events leading to the increase of cells with DNA content equal to or greater than 4c (Fig. 1b; Fig. 2).

**Fig. 3:**
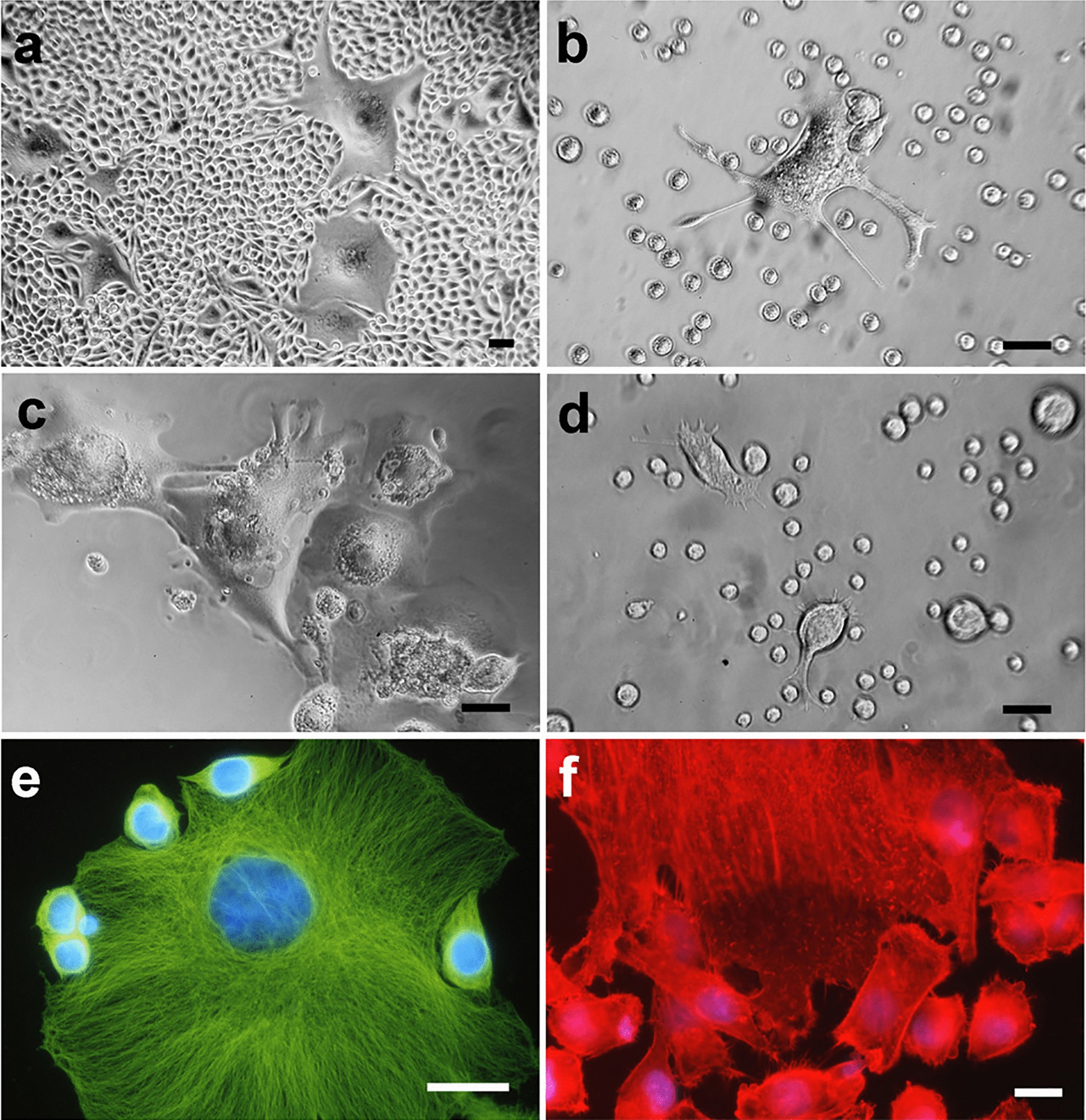
*Cell heterogeneity and adhesion variability.* Phase contrast micrographs (**a–d**) and fluorescence microscopy (**e**, **f**) of PEG-treated NIHs cells. In **a**, the morphological aspect of the culture before trypsinization; in **b** and **c,** the effects of trypsin action for 7 min are shown: after this incubation time, cells with large dimensions are still adherent, while the small cells are in suspension. In **c**, an area of the growth substrate showing the persistence of large cells after trypsinization for 7 min is shown. In **d**, it is possible to observe the detachment of large cells after 15 min of trypsinization. In **e** and **f**, immunofluorescence techniques allow us to evaluate cytoskeleton organization in giant cells: **e**, microtubules; **f**, actin microfilaments. Scale bar in **a**: 40 µm; in **b** and **d**: 10 µm; in **c**, **e**, and **f**: 20 µm

After one trypsinization (following 30 h of permanence in PEG-free complete medium after PEG-treatment) and five trypsinizations of NIHs cultures, DNA content histograms (Fig. 1c, d), respectively) and phase contrast micrographs (inserts in Fig. 1c, d) show the loss of giant cells, with very large dimensions. In general, culture splitting caused a decrease and/or the disappearance of several subsets of PEG-induced aneuploid/polyploidy cells; this effect can be partially due to cell dilution during culture propagation (Figs. 1, 2). After one trypsinization, the microfluorometric analysis showed a tendency to reduce cell subpopulations with DNA content greater than 4c, including new main giant cell subsets with 64c, 110c, and 140c DNA content (Fig. 1c; Fig. 2). After trypsin incubation, the persistence of cells with larger dimensions in both flasks and coverslips appeared to be related to their strong adhesion to the growth substrate and high cytoskeletal organization. These cells detached from the substrate after trypsin incubation for 15 min, while cells with small and intermediate dimensions detached more easily after trypsin incubation for 7 min (Fig. 3). After the fifth trypsinization, microfluorometric and morphological analyses performed following PEG treatment (Fig. 1d) indicate that, compared to one trypsinization (Fig. 1c), NIHs cultures are further impoverished in polyploid cells with DNA content ranging from 4c to − 24c. Giant cells with DNA content greater than 24c disappear.

### Mononuclearity, multinuclearity and nuclear anomalies before and after PEG treatment

As reported in the Methods section, during DNA microfluorometric measurements (Hoechst 33,342 and acridine orange fluorochromization), images of the different nuclear conditions in individual cell population were acquired. Thus, the obtained photomicrographs allowed us to correlate, in the same cells, the DNA content to the different features, such as number, size, positioning of the nuclei, and the eventual presence of anomalies such as micronuclei, nuclear envelope budding, and internuclear bridges. An example of their percentage distribution in the different experimental conditions is shown in Table 1.

**Table 1:** Mononuclearity, multinuclearity and main nuclear anomalies

	Small			Intermediate				Large		
	4c	6c	8c	10c	12c	16c	24c	64c	110c	140c
**Mononuclearity**										
Untreated	96	63	39	48	41	13	22	ND	ND	ND
PEG	88	67	26	33	29	7	16	0	0	0
VBL	70	ND	18	51	35	9	13	ND	ND	ND
PEG-VBL	73	ND	24	52	48	21	25	ND	0	ND
**Multinuclearity**										
Untreated	4	37	61	52	59	87	78	ND	ND	ND
PEG	12	33	74	67	71	93	84	100	100	100
VBL	30	ND	82	49	65	91	87	ND	ND	ND
PEG-VBL	27	ND	76	48	52	79	75	ND	ND	ND
**Small Micronuclei**										
Untreated	0	65	39	26	30	14	8	ND	ND	ND
PEG	0	63	36	22	27	15	7	0	0	0
VBL	0	ND	0	0	ND	0	ND	ND	ND	ND
PEG-VBL	0	ND	0	0	0	0	0	0	ND	ND
**Nuclear budding and fragmentation**										
Untreated	0	0	0	2	4	9	12	ND	ND	ND
PEG	0	0	0	2	6	13	16	0	0	0
VBL	0	0	0	3	10	17	ND	ND	ND	ND
PEG-VBL	0	0	0	3	10	19	21	ND	ND	ND
**Internuclear bridges**										
Untreated	0	2	0	4	8	7	6	ND	ND	ND
PEG	0	1	1	4	4	6	8	0	0	0
VBL	0	0	0	0	0	0	0	ND	ND	ND
PEG-VBL	0	0	0	0	0	0	0	ND	ND	ND

Example of percentage distribution of mononuclearity, multinuclearity and main nuclear anomalies in polyploid/aneuploid NIHs cells. The individual percentage values were calculated within the subpopulations with different DNA content. The nuclear features examined could occur simultaneously in the same cell. Changes of nuclear conditions were evaluated in both controls (PEG-untreated cells) and after PEG treatment. Vinblastine incubation was carried out in both PEG-untreated cells (VBL) and in previously PEG-treated cultures (PEG-VBL). To establish the percentage of various nuclear features, at least 200 cells for each DNA content value (c) were evaluated. ND: the value was Not Determined due to the absence of cells with the respective DNA content

The presence of a single nucleus was observed in cells with DNA content values up to 24c. Polyploid mononucleated NIHs cells have shown an oscillating trend in the different cellular subpopulations and in relation to the different experimental conditions (Table 1).

**Fig. 4:**
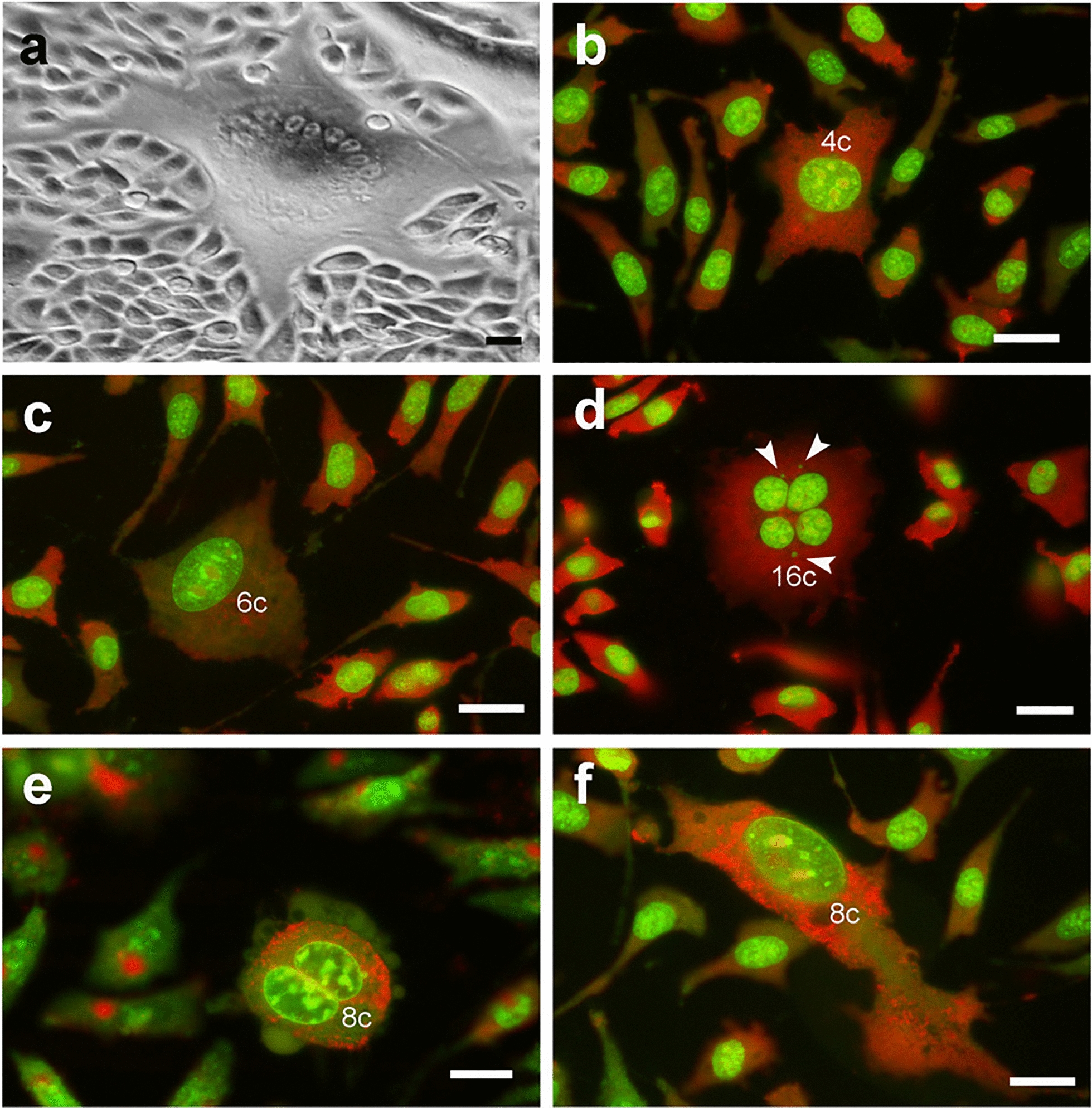
*Cell mononuclearity and multinuclearity related to the DNA content.* Phase contrast (**a**) and acridine orange (AO) fluorescence (**b**–**f**) micrographs of PEG-treated NIHs cells. In **a,** the morphological aspect of the culture, with a multinucleated giant cell. In **b**, **c**, **d**, and **f**, AO staining, performed according to a standard protocol, allowed us to observe green nuclear DNA and red cytoplasmic RNA fluorescence. In all fluorescence micrographs, the value of the DNA content of polyploid cells is reported. In **d**, arrowheads indicate micronuclei in the cytoplasm of a 16c tetranucleated cell, while in **e**, the punctuate red fluorescence of acidic vesicles in the cytoplasm (after the use of dilute AO solution) is shown. Scale bars: 10 µm

**Fig. 5:**
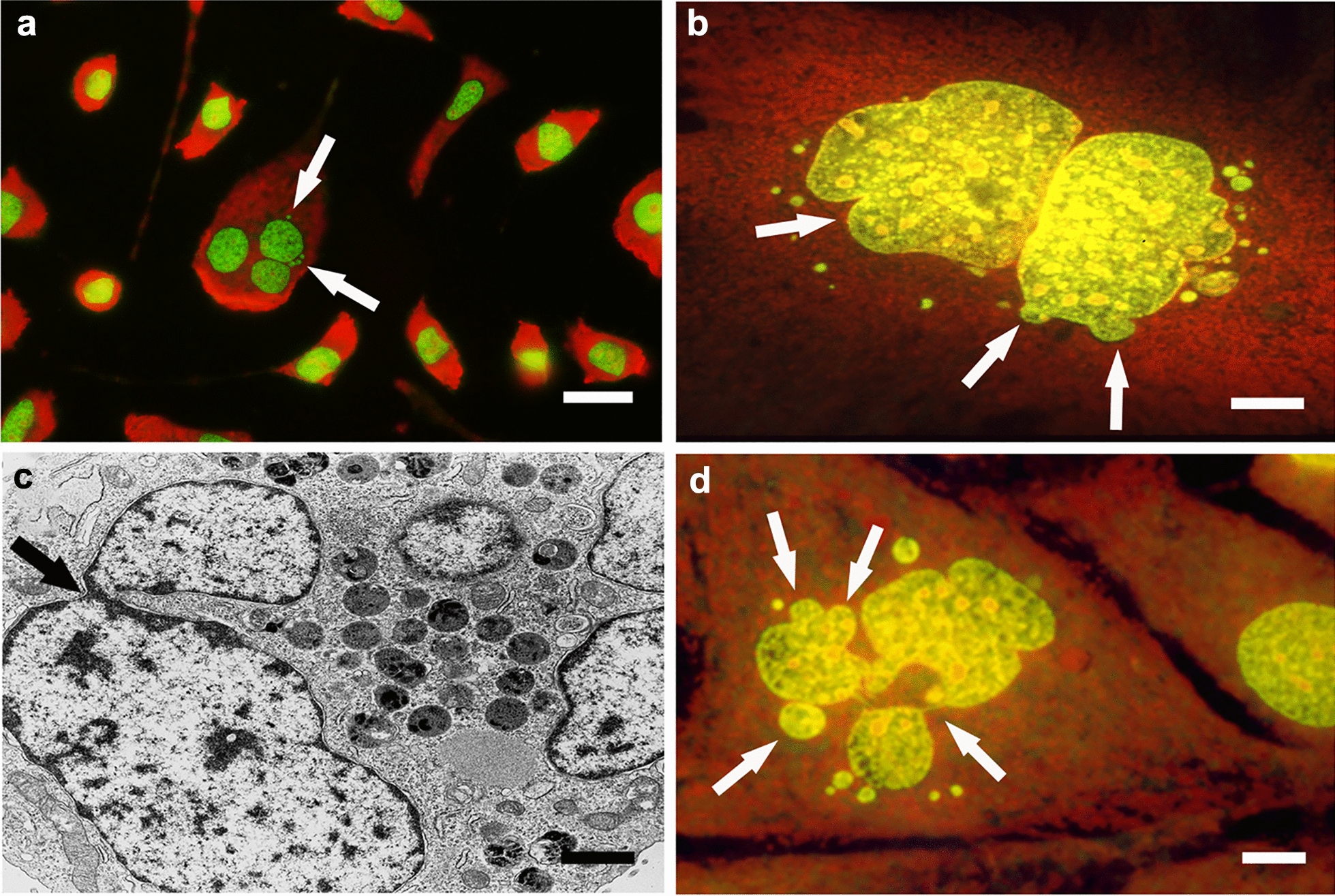
*Changes and abnormalities of cell nuclei.* Acridine orange (AO) fluorescence (**a**, **b**, **d)** and TEM (**c)** micrographs of PEG-treated polyploid NIHs cells. AO staining, performed according to a standard protocol, allowed us to observe yellow/green nuclear DNA and red cytoplasmic RNA fluorescence. In **a**, a trinucleated cell with micronuclei in the cytoplasm (arrows). In **b**, **c**, and **d**, events of nuclear envelope budding (arrows) resulting in the detachment of nuclear portions and the presence of micronuclei with heterogeneous sizes are shown (**b**, **c**, and **d)**. The ultrastructural image in **c** shows a situation similar to that shown in **d**. The arrow in **c** indicates a possible detachment of a nuclear portion. Scale bars in **a**: 10 µm, in **b** and** d**: 5 µm, in **c**: 1 µm

**Fig. 6:**
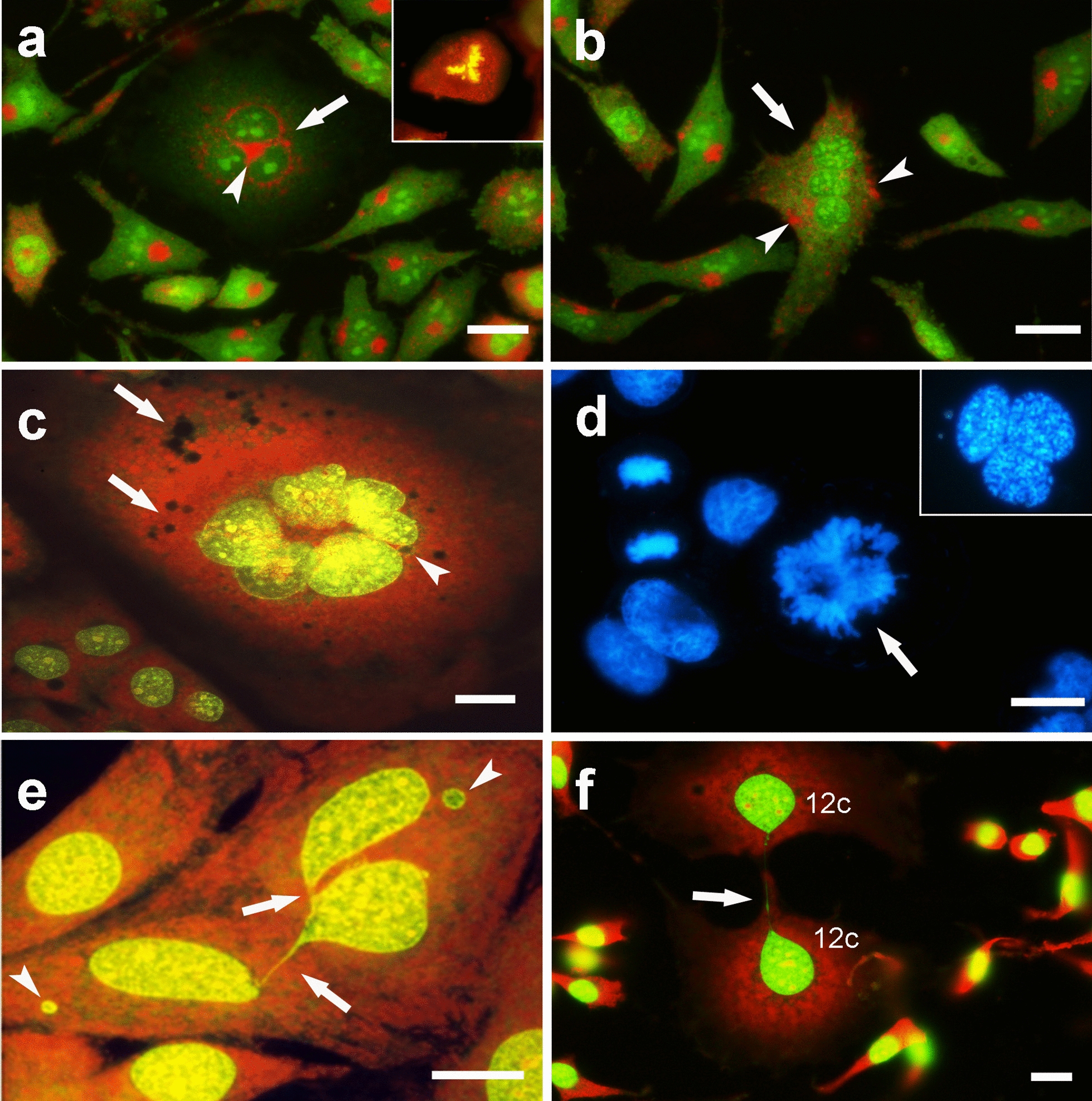
*Nuclear dynamics related to cell division processes*. Acridine orange (AO) fluorescence (**a**, **b**, **c**, **e**, **f)** micrographs of PEG-treated NIHs cells. In **a** and **b**, the punctuate red fluorescence of acidic vesicles after use of dilute AO solution is shown; in this staining condition, nuclei are marked with a weak green fluorescence. The arrows in **a** and **b** indicate trinucleated cells in which the distribution of aggregates of red puncta allows us to deduce the positioning of the Golgi area that appears to be mainly in the center of the cell in **a** (arrowhead) or more dispersed in aggregates in the cytoplasm in the cell in **b** (arrowheads). In the insert in **a,** tripolar mitosis is shown. In **c**, **e**, and **f**, AO staining, performed according to a standard protocol (see Methods section), allowed us to observe green nuclear DNA and red cytoplasmic RNA fluorescence. In the hexanuclear 24c cell in **c**, the arrows indicate areas of cytoplasm discontinuity; the arrowhead indicates a micronucleus. In **e** and **f**, chromatin internuclear bridges are visible after cytokinesis process completion; arrowheads in **e** indicate micronuclei. In **d**, Hoechst 33,342 fluorescence in a microscopic field with irregular tripolar mitosis (arrow), in the insert, a trinucleated cell with single nuclei synchronized in prophase is shown. Scale bars in **a**, **b**, **c**, and** f**: 10 µm, in **d** and **e**: 5 µm

Excluding cells with 2c DNA content, multinuclearity was detected in all the main polyploid subpopulations constituting the NIHs culture and was observed as the prevalent condition in polyploid cells with DNA content equal to or greater than 8c. Data on mononuclearity and multinuclearity are reported in Table 1, while Fig. 4 shows the morphology of mononucleated and multinucleated cells with different DNA contents. In detail, 4c and 6c cells (small elements of the 4–8c range) were detected in the prevailing mononucleation condition (Fig. 4b, c). Alternately, 4c and 6c cells showed the presence of two or three nuclei, respectively. Cells with 8c DNA content were detected as both binucleated (Fig. 4e) and mononucleated (Fig. 4f). In trinucleated cells, different situations in terms of the positioning of nuclei and their DNA content were found. In some cases, nuclei showed an unbalanced (from 2.8c to 4.4c for each nucleus) DNA content (total DNA per cell ranging from 10 up to hypo-12c); in other cases, the microfluorometric analysis indicated trinucleated cells showing different combinations of nuclei with 2c or 4c DNA content. This latter situation can be considered the outcome of three-cell fusion according to the following combinations: three 2c cells (total DNA content 6c); two 2c cells and one 4c cell (total DNA content 8c); two 4c cells and one 2c cell (total DNA content 10c); and three 4c cells (total DNA content 12c). Considering the positioning of nuclei, two types of trinucleated cells were detected. In the first situation, trinucleated hypo-12c cells showed nuclei arranged according to the vertices of a triangle (Figs. 5a, 6a). As reported in the discussion section, they can be the result of tripolar mitoses ) (insert in Fig. 6a, d) without cytokinesis occurring in mononucleated 6c cells. In trinucleated cells derived from this kind of event, nuclei can retain the ability to enter mitosis, as evidenced by events of prophase synchronization that have been detected   (insert in Fig. 6d); frequently, these cells may have one or more micronuclei dispersed in the cytoplasm (Fig. 5a).

In the second situation, trinucleated cells exhibit a different positioning of nuclei: they do not appear to be arranged according to the vertices of a triangle but rather aligned (Fig. 6b). Microfluorometric measurements indicated that these trinucleated elements may be the result of 2c and 4c cell fusion, according to the different combinations considered above. In these cells, micronuclei were not detected. In both types of trinucleated cells, acridine orange staining, carried out under suitable conditions to identify the acidic vesicles in the cytoplasm (see Methods section), allowed us to deduce a different subcellular localization of the Golgi apparatus area (Fig. 6a, b). Based on this criterion, two types of localization of the Golgian area can be identified: more concentrated in a single area in the center of the cell (Fig. 6a) or more dispersed in the cytoplasm (Fig. 6b). In addition to being trinucleated, 12c cells were also detected as mononucleated. In some cases, chromatin internuclear bridges, due to cell division anomalies, were observed between mononucleated 12c cells (Fig. 6f). Among the cells with intermediate dimensions, in addition to the 10–12c cells, 16c and 24c cells were also detected. As previously reported, all these cell subpopulations increased in percentage after PEG treatment compared to the control (Fig. 2).

The 16c cells were detected in the conditions of binucleation and tetranucleation, with two 8c nuclei and four 4c nuclei (Fig. 4d), respectively. The 24c subpopulation was found to consist of mononucleated and hexanucleated cells (Fig. 6c). In some multinucleated 24c cells, it was possible to detect small micronuclei that could arise from unsegregated chromosomes derived from mitosis without cytokinesis of trinucleated 12c cells (24c after the S phase). These micronuclei may contribute, as well as chromatin bridges, to the imbalance of the DNA content of the individual nuclei and the genesis of an aneuploid state. Internuclear bridges have also been observed following cell division processes based on tripolar mitosis (Fig. 6e).

Especially in polyploid cells with intermediate dimensions (10–24c DNA content), multinuclearity was frequently accompanied by different nuclear anomalies (Table 1), such as small micronuclei (Fig. 4d; Fig. 5a) and nuclear envelope evaginations resulting in nuclear portion detachment leading to micronuclei formation of heterogeneous size (Fig. 5b, c, d).

The 64c, 110c, and 140c subpopulations were found to consist exclusively of multinucleated cells. In these very large giant elements, a number of nuclei between 30 and 50 was determined (Fig.  4a); microfluorometric measurements indicated that single nuclei displayed 2c or 4c DNA content. The presence of these cells can be referred to as PEG-induced fusion.

### Nuclear and cytoplasmic modifications and lysosomal and autophagic activity of untreated and PEG-treated NIHs cells

**Fig. 7:**
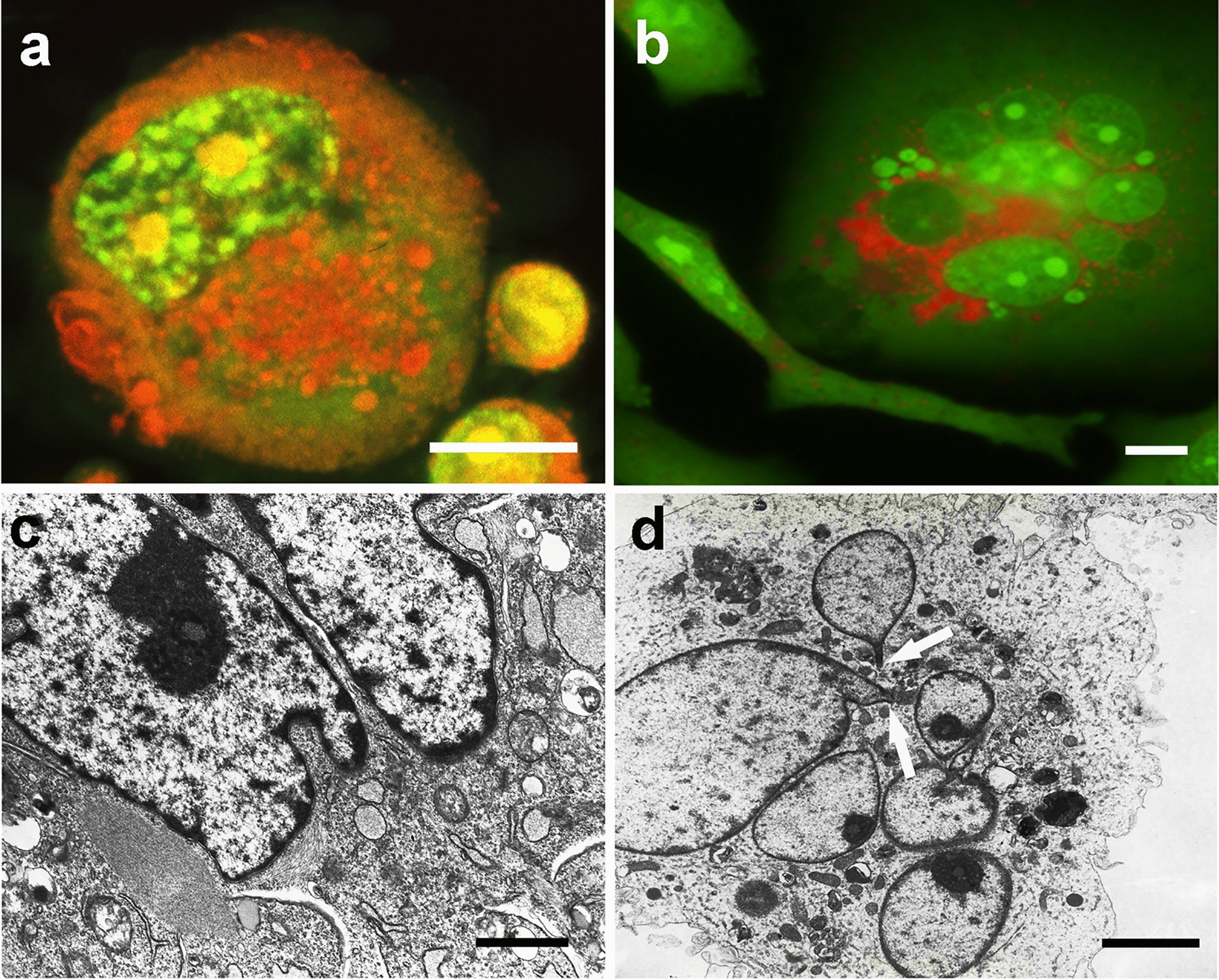
*Degenerative aspects of cytoplasm and nuclear changes.* Acridine orange (AO) fluorescence (**a, b)** and TEM (**c**, **d)** micrographs of PEG-treated polyploid NIHs cells. Images in **b** and **d** refer to cultures subsequently treated with vinblastine. It is possible to observe diffuse granular red fluorescence (AO) or the presence of vacuoles, secondary lysosomes, and residual bodies in the cytoplasm (TEM). These aspects, indicative of autophagic events in the cytoplasm of polyploid/aneuploid cells, appeared to be correlated with changes in the nuclear morphology (**c**), presence of chromatin aggregates (**a)**, and fragments/micronuclei of heterogeneous size (**b**, **d)**. In the image in **d**, the arrows indicate chromatin bridges that appear to connect the fragments to the remnant of the main nucleus. Scale bars in **a** and **b**: 5 mm; in **c**: 1 mm; in **d**: 2 µm

**Fig. 8:**
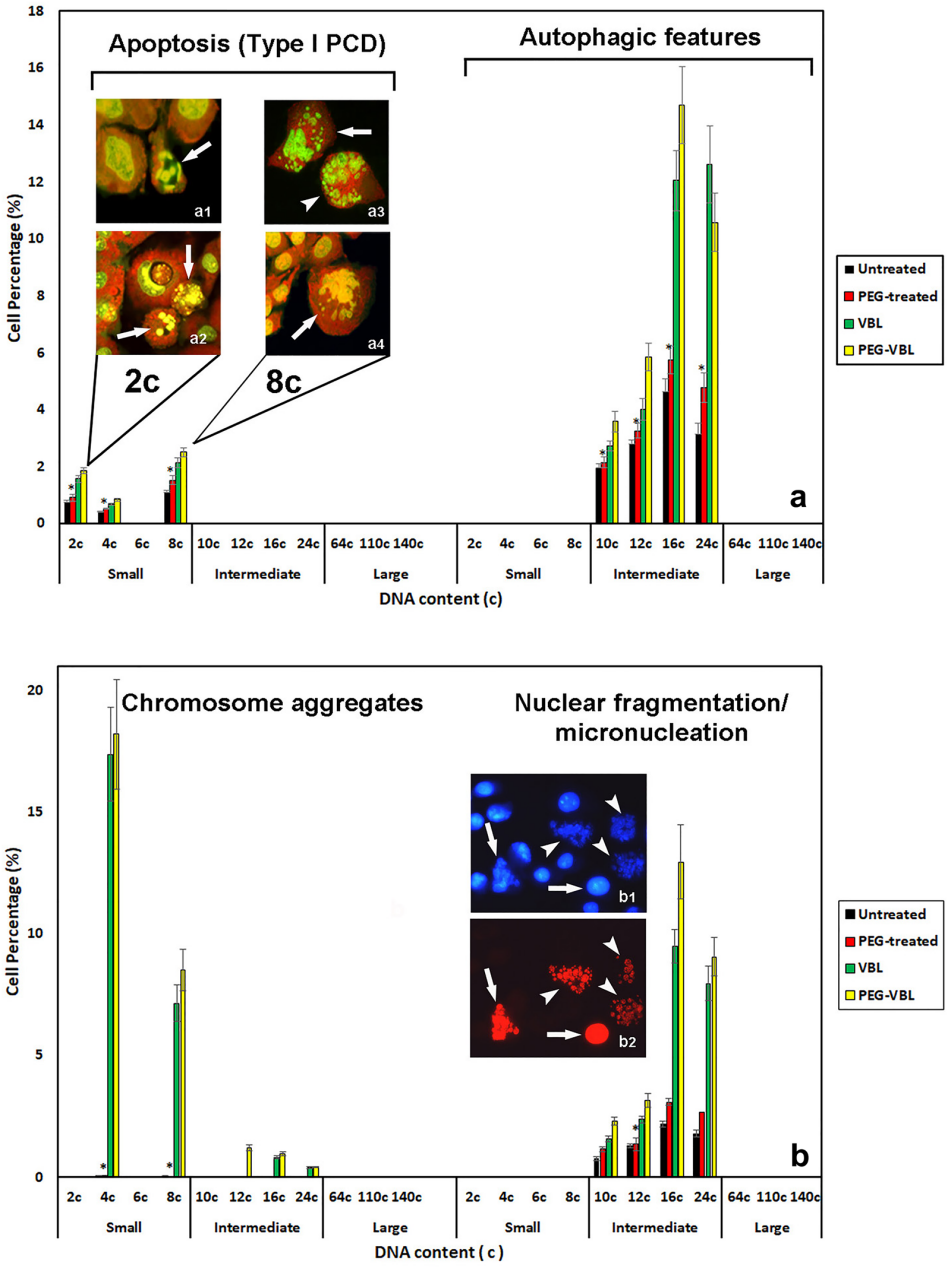
*Cell degeneration events.* In **a**, percentage distribution of apoptotic programmed cell death events (type I—PCD) and a form of cell degeneration with autophagic features. Morphological examples of apoptotic death in cells with 2 c and 8 c DNA content are shown in the inserted fluorescence micrographs (acridine orange staining). In **a1** and **a2**, the arrows indicate chromatin condensation with margination against the nuclear envelope and karyorrhexis events, respectively. In **a3** and **a4**, the arrows indicate nuclear apoptotic-like alterations (shrinkage and nuclear budding). In **a3,** an example of nuclear fragmentation/micronucleation can be observed in a cell with a DNA content near the 12 c value (arrowhead). In **b**, the percentages of chromosome aggregates and nuclear fragmentation/micronucleation are shown. The inserted fluorescence micrographs (**b1** and **b2**) show, in a microscopic field (HO 33342-blue fluorescence) (**b1**), the nuclear positivity to the TUNEL reaction (red fluorescence) (**b2**), indicating DNA fragmentation in apoptotic nuclei (arrows) and alternative forms of cell death (arrowheads). The individual percentage values were calculated within the subpopulations with different DNA content. The various cellular aspects were evaluated in both controls (PEG-untreated cells) and after PEG treatment. Vinblastine incubation was carried out in both PEG-untreated cells (VBL) and in previously PEG-treated cultures (PEG-VBL). To establish the various cellular aspects, at least 300 cells for each DNA content value were evaluated. The values, which refer to the main cell subpopulation of NIHs culture in the different experimental conditions, represent the mean number of cells ± SEM of triplicate morphological evaluations. The asterisks (*) indicate the values, referred to the different experimental conditions, that are not significantly different (P > 0.05 after Student's t-test) from the control (PEG-untreated cells). Type I PCD values, referring to the 4c and 8c subpopulations, are all significantly different (P < 0.05 after Student's t-test) from those of the 2c subpopulation

As reported in the Methods section, the use of acridine orange under cell viability conditions allowed us to evaluate lysosomal and autophagic activities. After staining of PEG-untreated and PEG-treated cultures, in intermediate polyploid cells (10–24c DNA content), changes in nuclear morphology, including the presence of chromatin aggregates (Fig. 7a) and fragments/micronuclei of heterogeneous size (Fig. 7b), appeared to be associated with the presence of bright red granulations, indicative of autodigestive events (Fig. 7a, b). The percentage of cells with autophagic features under the different experimental conditions is shown in Fig. 8.

Regarding the different subpopulations of polyploid intermediate cells, no significant changes in the percentage of autophagic events before and after PEG treatment were found. TEM microscopy confirmed that cytoplasmic degenerative processes occur in cells exhibiting nuclear anomalies; images showing comparable situate, ions were obtained by fluorescence and TEM microscopy 6 (Fig. 7b vs d). These features, in part referable to autophagic cell death, detected in both control conditions and after PEG treatment, occurred more frequently after VBL incubation of NIHs culture (see below), both before and after PEG treatment ( Fig. 8).

### Effects of VBL incubation before and after PEG treatment

#### DNA content analysis and microscopic observations at the nuclear level

**Fig. 9:**
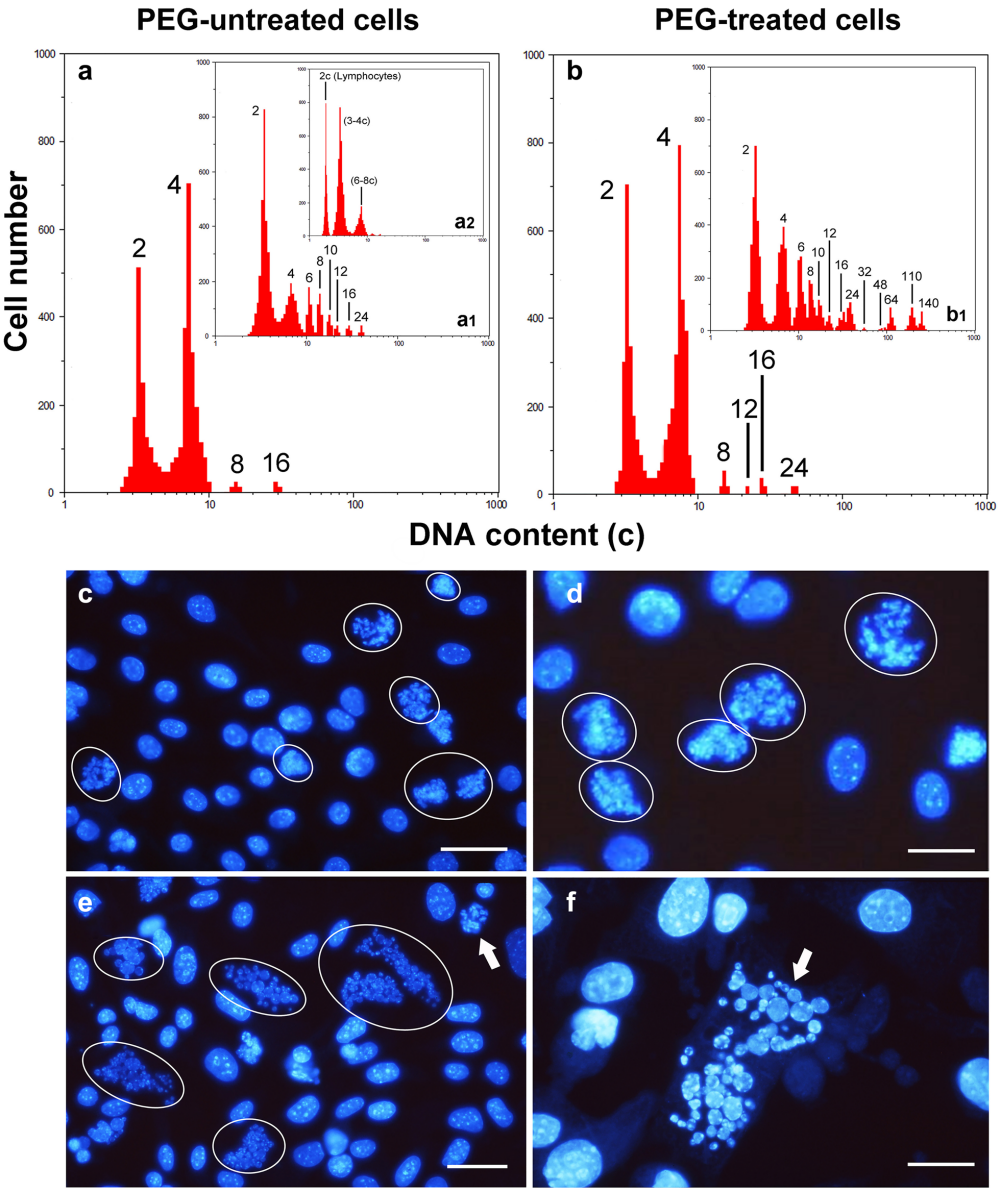
*Cytokinetic and nuclear morphology changes induced by vinblastine.* Typical microfluorometric analyses of the DNA cellular content (**a**, **b**) and Hoechst 33342 fluorescence micrographs (**c**, **d**, **e**, **f**) after VBL incubation in PEG-untreated cells (**a**, **c**, **e**) and PEG-treated cells (**b**, **d**, **f**). Insert **a1** in **a** and insert **b1** in **b** represent microfluorometric measurements in the absence of vinblastine. Insert **a2** in **a1** represents a microfluorometric measurement in NIH/3T3 cell line before serum starvation; the first peak (3c–4c DNA content) in the abscissa axis of microfluorimetric histogram was arbitrarily indicated as 2c. In **c** and **d**, microscopic fields particularly rich in 4–8c aggregates of chromosomal structures (circles in **c** and **d**), as a consequence of the mitosis blockade, can be observed. In **e** and **f**, nuclear events characterized by large micronuclei of heterogeneous dimensions (circles in **e** and arrow in **f**) are reported. The arrow in **e** indicates chromosomal structures. Scale bars in **b** and **c**: 10 µm; in **e** and **f**: 5 µm

VBL incubation was performed in NIHs cells before and after PEG treatment. In both cases, the drug induces a remarkable simplification of the cytofluorometric profile (Fig. 9a, b). Some cell subpopulations disappear (see also Fig. 2) compared to the corresponding reference situations (Fig. 1a, b), which are also reported as inserts in (Fig. 9a, b), respectively.

In PEG-untreated culture, VBL induced a significant accumulation of cells with 4c DNA content as an effect of the action on the highly proliferating 2c subpopulation; among polyploid cells with DNA content greater than 4c, only 8c and 16c subpopulations persisted, even if reduced in percentage (see also Fig. 2). In PEG-treated cultures, VBL induces similar effects as regards the incidence of 4c, 8c, and 16c subpopulations (Fig. 2). In PEG-untreated cultures, 12c and 24c subpopulations are absent after incubation with VBL (insert in Fig. 9a; Fig. 2); instead, these same subpopulations persist in PEG-treated cultures, even if their percentage incidence decreases compared to the control condition (insert in Fig.  9b; Fig. 2). In PEG-treated culture, polyploid cells with DNA content higher than 24c disappeared, after VBL incubation (Fig. 9b, Fig. 2).

Fluorescence microscopy observations, after Hoechst 33342-DNA staining of PEG-untreated (Fig. 9c, e) and PEG-treated (Fig. 9d, f) NIHs cells, allowed us to assess the effects induced at the nuclear level by VBL incubation. In both PEG-untreated (Fig. 9c, e) and PEG-treated (Fig. 9d) cultures, aggregates of chromosomal structures (mainly derived from altered metaphases) unevenly distributed in the microscopic fields are identifiable. Microfluorometric measurements of these chromosomal aggregates mainly indicated a DNA content near 4c and 8c values; the highest percentage incidence was detected in the 4c subpopulations (Fig. 8). In a very limited number of chromosomal structure aggregates, a total DNA content close to 16c and 24c was measured. Chromosomal aggregates with a value near 12c were detected only in PEG-treated NIHs cultures before VBL incubation (Fig. 8).

Other nuclear modifications induced by the antimicrotubular poison were represented by the appearance of a high number of micronuclei per cell, with heterogeneous dimensions, in both PEG-untreated (Fig.  9e) and PEG-treated (Fig. 9f) NIHs cultures. In the aggregates of micronuclei, a total DNA content close to the 16c value, or included in the 16c-24c range, was prevalently detected. With a lower percentage incidence, the measurements also revealed a DNA content in the 10–12c range (Fig. 8). VBL action did not produce significantly different effects between untreated and PEG-treated cultures (Fig. 8).

The use of the TUNEL assay made it possible to detect that these heterogeneous micronuclei aggregates were positive for the reaction (Fig. 8b). After incubation with VBL, fluorescence microscopy, through DNA fluorochromization (acridine orange and Hoechst 33342 staining) and the TUNEL reaction, also allowed the detection of a low number of conventional apoptosis events (type I PCD) and only in 2c, 4c and 8c cells (Fig. 8a).

After VBL incubation, a low number of conventional apoptotic events was detected and only in 2c, 4c and 8c cells. In particular, typical features of type I apoptosis, such as chromatin hypercondensation and nuclear fragmentation, were observed with a higher incidence in 2c and 8c cells (Fig. 8a). The use of the TUNEL reaction made it possible to detect that in these apoptotic cells, the nuclear alterations were correlated with the positivity of the reaction and therefore with DNA fragmentation (Fig. 8b).

The low percentage of hypo-2c, hypo-4c, and hypo-8c values, due to the DNA loss of apoptotic cells, did not make it possible to identify them in microfluorometric DNA content histograms.

**Fig. 10:**
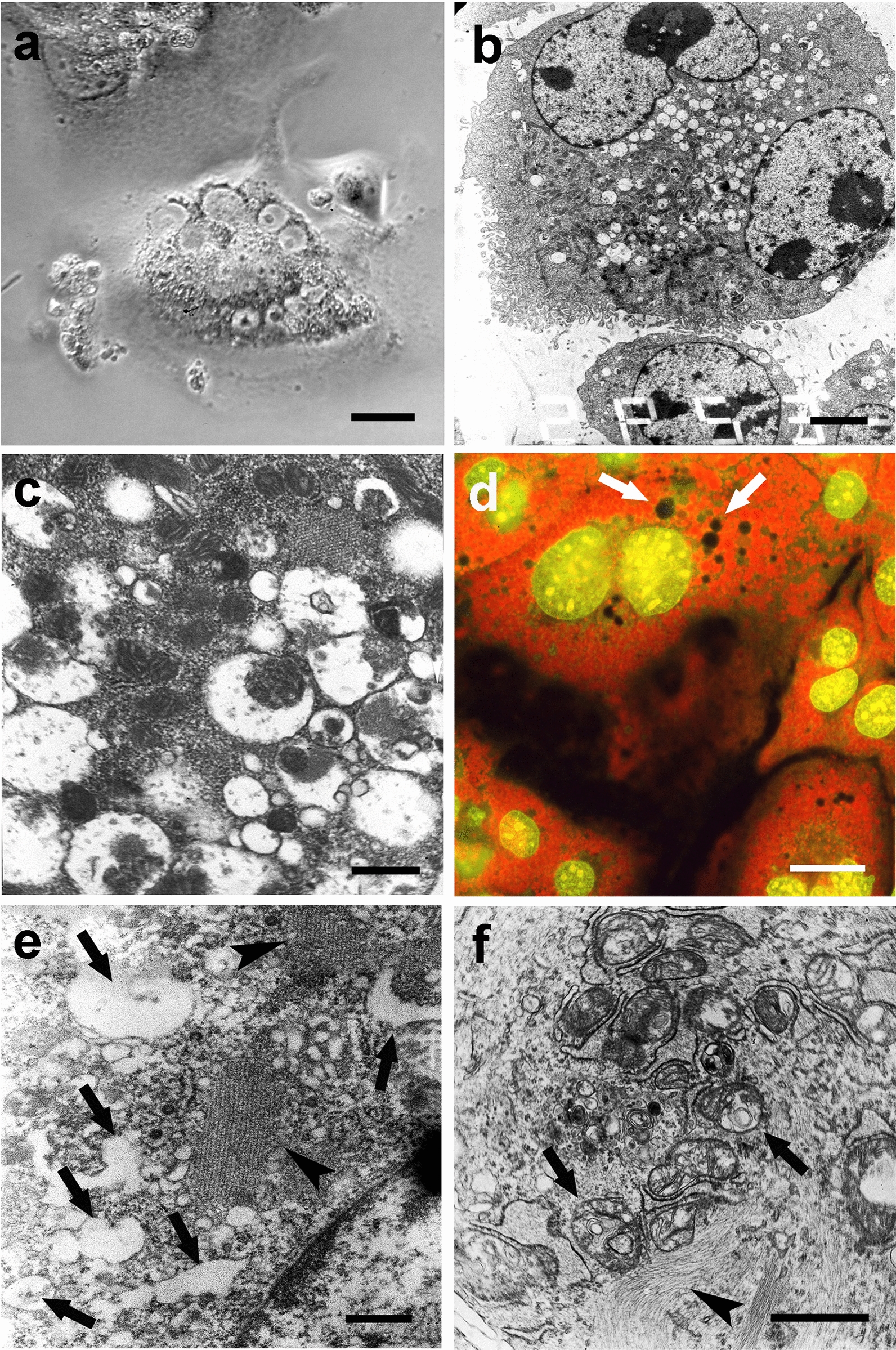
*Vacuolation of cytoplasm and cytoskeletal modifications.* Phase contrast (**a**), TEM (**b**, **c**, **e**, **f**), and acridine orange (AO) fluorescence (**d**) micrographs of PEG-treated NIHs cells. Images in **a**, **c**, **e**, and **f** refer to cells subsequently incubated with vinblastine. In **a** and **b**, signs of cytoplasmic changes in polyploid/aneuploid cells are visible as granulations (phase contrast micrograph of a multinucleate cell in **a**) and vacuolations (TEM micrograph in **b**). In **c**, a detail of the vacuoles that appear to be empty or with variable content. AO staining (**d**) allowed us to observe dark areas (arrows) in the cytoplasm of a polyploid/aneuploid cell. This aspect may be related to the images obtained by TEM microscopy, which allowed us to note electron-transparent areas of cytoplasmic discontinuity (arrows), especially in VBL-incubated cells (**e**). In **e** and **f**, VBL effects on the cytoskeleton can also be observed: in particular, in **e**, tubulin paracrystals (arrowheads) close to areas of extensive vacuolation (arrows) are visible. In **f**, a bundle of intermediate filaments (arrowhead) in proximity to a vacuolation area is evident. Vacuoles show heterogeneous content, which may result from the degradation of organelles or endomembranes (myelin figures indicated by arrows). Scale bars in **a** and **d**: 10 µm; in **b**: 1 µm; in **c** and **e**: 3 µm; in **f**: 1 µm

Polyploid cells with DNA content higher than 8c, in addition to the drastic modifications of the interphasic nucleus, increased by VBL action, showed evident modifications of the cytoplasm, indicative of autophagic process activation ) and (Fig. 8a). The occurrence of autophagic digestion events before and after the antimicrotubular poison action can be evaluated, even from an ultrastructural point of view, in Fig. 10 (see below for description).

### Cytoplasmic degenerative aspects of polyploid intermediate giant cells and ultrastructural details

As already described, AO fluorochromization allowed us to note that the drastic changes in NIHs cell nuclear morphology appear to be related to cytoplasm alterations, identifiable by the appearance of bright red granulations (Fig. 7a, b). These features, likely indicative of autophagic activity, occur more frequently in mononucleated and multinucleated intermediate cells and can also be detected through phase contrast microscopy as dark granulations in the cytoplasm (Fig. 10a). In these cells, TEM ultrastructural analysis showed the presence of cytoplasmic areas with vacuolation (Fig. 10b). The vacuoles usually contain residual bodies and myelin figures (Fig. 10c, f) and express autodegradation of organelles and endomembranes. In several cells with extensive vacuolation, the wide areas of cytoplasmic discontinuity (Fig. 10e) may be derived from the possible confluence of smaller-size vacuoles that appeared to be empty. Features of the same type can correspond to dark and nonfluorescent areas detected at the cytoplasm level after AO fluorochromization (Fig. 10d). These dark vacuolizations (also shown in Fig. 6c) are predominantly observed in the cytoplasm of polyploid cells.

**Fig. 11:**
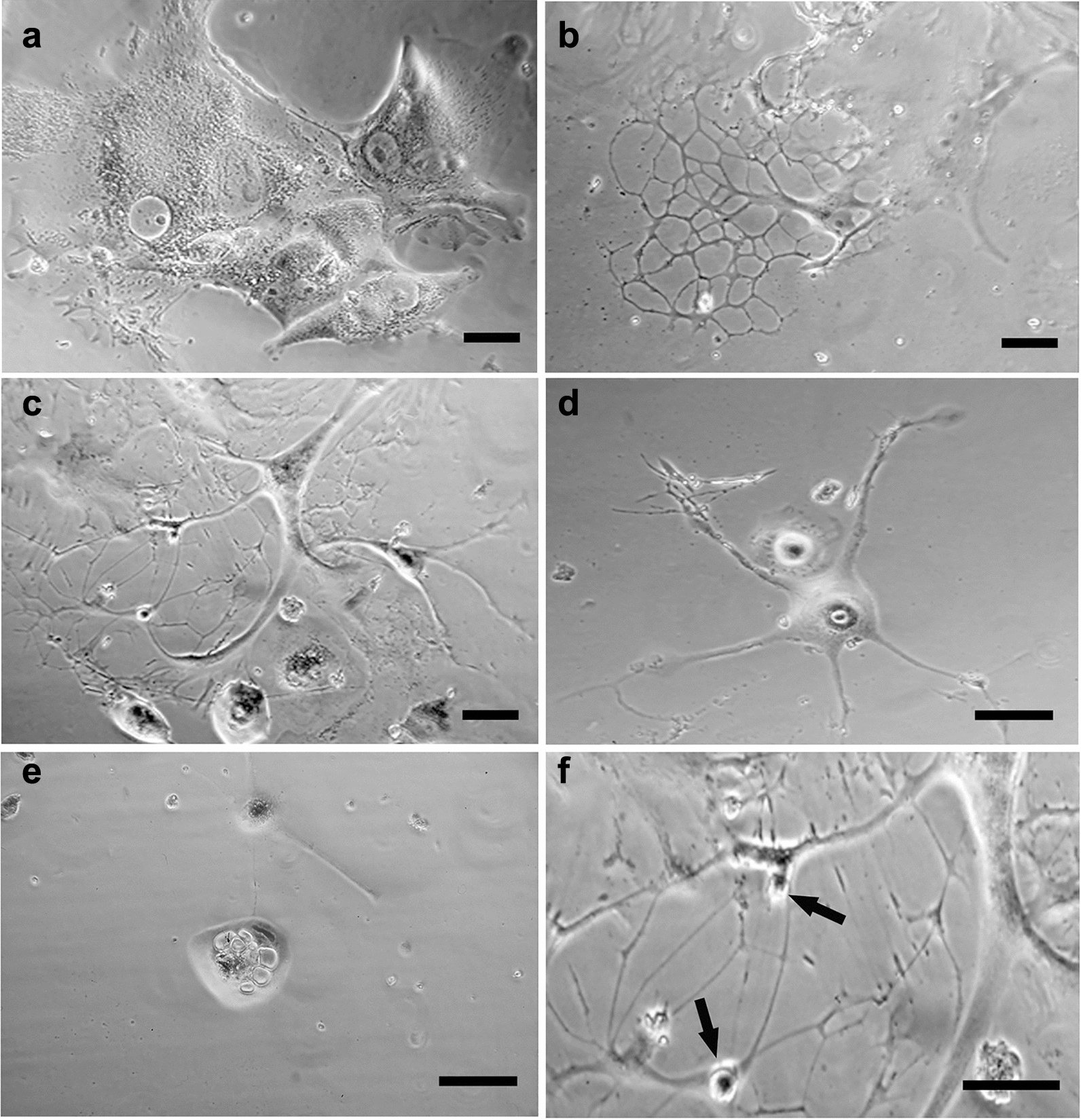
*Possible pathway of degeneration and detachment from the substrate of polyploid cells.* Phase contrast micrographs of PEG-treated NIHs cells. The images refer to the possible progression steps (**a**, **b**, **c**, **d**, **e**) of degenerative events in polyploid/aneuploid cells, resulting in detachment from the growth substrate. In **a**, middle/large cells with an intense cellular granularity (that correlates with the high degree of autophagic vacuolations detected in this type of cell through TEM microscopy) are visible. In **b**, it is possible to observe the cytoplasm restricted to a thin network supported by subtle and long cytoplasmic expansions. This aspect could be the possible consequence of drastic cytoplasmic remodeling partly due to self-digestive cytoplasmic events. In the final stages of the process, these cells, which would acquire a neuron-like morphology (**d**), can retract their cytoplasmic extensions by detaching from the substrate and assuming an apoptotic-like morphology (**e**). In **f,** a detail of a neuron-like cell is reported: the arrows indicate possible budding events on thin extensions with the release of daughter cells with low ploidy levels. Scale bars in **a**, **b**, **c**, **d**, and **e**: 15 µm; in **f**: 30 µm

After VBL incubation, the signs indicative of autophagic processes appeared to be increased, especially in 16c and 24c cells (Fig. 8), and in some cases, vacuolations appeared to be close to tubulin paracrystals. These structures, which consist of packed microtubules, can be observed in longitudinal sections in transmission electron micrographs (Fig. 10e) As a possible reflection of microtubular network alterations, intermediate filaments tend to form compact bundles (Fig. 10f). The cytoplasmic modifications reported above could be implicated in detachment from the substrate and in degeneration, detected by phase contrast microscopy, in some intermediate/large polyploid cells. The confluence of discontinuous cytoplasmic areas and cytoskeletal reorganization could lead to drastic cytoplasmic remodeling before cell detachment. A possible sequence of the events involved is depicted in Fig. 11 in a set of micrographs, which refers to PEG-treated NIHs cells. The first phase of the degenerative process could be represented by the appearance of diffuse cytoplasm granularity observed by phase contrast microscopy (Fig. 11a). This cellular aspect may be correlated with the increase in vacuoles detected through TEM microscopy (Fig. 10b). During the progression of the process, the possible confluence of the vacuoles and the contribution given by autodigestive phenomena could lead to the reduction of cell mass with the cytoplasm that appears restricted to a thin network, supported by subtle and long cell expansions (Fig.  11b, c). In the following steps, these cells, which would acquire a neuron-like morphology (Fig. 11d), can retract their cytoplasmic extensions by detaching from the substrate and assuming an apoptosis-like morphology (Fig. 11e). In some cases, careful observation by phase contrast microscopy allowed us to note budding phenomena on the thin extensions in cells with a DNA content of approximately 64c (Fig. 11f). As reported in the discussion section, these dynamic processes could lead to the genesis of low ploidy daughter cells.

## Discussion

This work records the potentially significant roles played in vitro by polyploid cells with low, intermediate, and high DNA content in the genesis and dynamics of subpopulations of NIHs fibroblastic culture before and after incubation with the fusing agent PEG. Referring to the complexity and heterogeneity of the cell populations in some tumors, this model can provide discussion elements on the events of appearance/disappearance and the fate of polyploid cells, evaluating the contribution of mitotic anomalies and cell fusion in these dynamics.

In NIHs cell subpopulations, the different DNA content was related to mononuclearity or multinuclearity and to cell persistence and cell loss dynamics. Therefore, in addition to the cytokinetic aspects, the role of degeneration and death processes was evaluated.

The study, performed in untreated and PEG-treated cultures in the absence of and after VBL incubation, can provide a basic contribution to defining, from morphological and cytokinetic points of view, models for preclinical investigation assays for the assessment of anticancer agent properties and activity. This appears to be relevant, especially in relation to the chemotherapeutic treatment of tumors with heterogeneous cell populations, including polyploid and aneuploid giant cells. Polyploidy can represent a transition stage toward aneuploidy, and by achieving these biological conditions, the cells of some tumors can become particularly aggressive and difficult to treat from a pharmacological point of view [15, 18]. Programmed cell death induction may represent a physiological mechanism useful for their selective elimination and can appear with variable features or alternative pathways to canonical apoptosis. Studies in the last two decades have demonstrated that programmed cell death, originally described as a substantially univocal biological event, can appear with some variability related to different biological situations [30, 31]. Data from our work demonstrate that in NIHs culture, polyploid cells, with intermediate dimensions (10c—24c), can die, even spontaneously, through degenerative mechanisms, alternative to canonical apoptosis, contributing to their transient decrease. Normally, during the routine maintenance of long-term cultures, the NIHs fibroblastic population shows, with some variability, the appearance/disappearance of polyploid elements with DNA content ranging from 4 to 24c. As in other cell cultures of different origins, the presence of polyploid/aneuploid cells can be related to repeated mitosis with cytokinesis failure and/or cell fusion. Genetic stability is maintained, in addition to the right functioning of DNA repair mechanisms, by coordinating the regulation of chromosome segregation and cytokinesis. Perturbations of this delicate balance can lead to anomalies of the cell division process, characterized by aberrant mitoses and/or formation of polyploid/aneuploid mono- and multinucleated elements, as a consequence of cytokinesis lack. These anomalies can alter normal cellular functions and contribute to neoplastic transformation or can cause mitotic catastrophe events [32, 33]. In general, little attention has been given to the relationship between alterations in cell division processes or cell survival capacity and the DNA content of polyploid/aneuploid cells. In NIHs culture, under control conditions, phase contrast microscopy allowed us to routinely observe that the increase in intermediate/large cells with polyploid DNA content is correlated with the state of cell confluence. Therefore, a contribution to their origin could be provided by cell fusion events favored by close cellular contacts. On the other hand, in subconfluent cultures, the presence of polyploid cells could also be due to abnormalities of the cell division mechanisms. Microflurometric measurements of DNA content in the NIHs control (PEG-untreated cells) showed the presence of polyploid elements with values ranging from 4 to 24c. In comparison with the control condition, PEG treatment, here used, allowed us to obtain an increase in polyploid cells in this range and the appearance of large plurinucleated cells with DNA content higher than 24c. Furthermore, the subconfluence condition permitted the maintenance of some proliferative capability of the culture and, at the same time, rent the cells (especially in some areas of the growth substrate) near enough to allow contact for PEG-induced fusion. In this way, it was possible to obtain polyploid cells with different origins, such as mitotic anomalies and cell fusion.

The interpretation of the data obtained in this work leads us to believe that the presence of intermediate cells (with 10-12c, 16c, and 24c DNA content values) may be, in part, the result of cytokinetic phenomena involving trinucleated and tetranucleated cells that can appear in the culture as a consequence of mitotic anomalies or cellular fusion events. Within intermediate cells, 12c cells appear with some frequency as trinucleated and characterized by variable situations. In addition to cells with three aligned nuclei (each with 4c DNA content) probably derived from fusion events, in some cases, trinucleated cells with values ranging from 2.8c to 4.4c for each individual nucleus were measured. This cell fraction can show total hypo-12c values (ranging from 10 to 12c) per cell, indicating a possible origin alternative to cellular fusion. In addition to the analysis of the DNA content of each nucleus, the distribution of the Golgi apparatus area could also provide indications to clarify trinucleated cell provenance. In elements with hypo-12c DNA content, the organelle appears to be positioned in the center of the cell, inside a triangle at whose vertices the nuclei are found. The imbalance of nuclear DNA contents could arise from tripolar mitoses, not accompanied by cytokinesis, occurring in 6c mononucleated cells. These cells, which should reach the 12c value after DNA duplication, actually show hypo-12c values. The imbalance of DNA content (not 4c) for a single nucleus can be related to unequal segregation of the chromosomes in telophase and/or to their partial nonsegregation with the formation of small micronuclei, observed with a higher frequency in these cells. The total hypo-12c value could be linked to the fate of micronuclei. These extranuclear chromatin bodies, surrounded by a nuclear envelope, may persist in the cytoplasm or may be reincorporated into the main nucleus or degraded and extruded from the cell [34] with a total DNA content decrease in these last cases. Alternately, in the 12c trinucleated cells showing aligned nuclei, probably originating from fusion events, small micronuclei are usually not detected. In these cells, the Golgi apparatus appeared to be dispersed in the cytoplasm rather than positioned in a single central area. On the other hand, the combined microfluorometric and microscopic analyses allowed us to detect that trinucleated cells with nuclei arranged as a triangle can express the ability to enter subsequent mitosis, as demonstrated by the presence of three mitotic nuclei synchronized in prophase.

Cells with DNA content close to or equal to 12c could play a role in the genesis of polyploid elements with a higher DNA content. Mitotic events, in the absence of cytokinesis, starting from trinucleated or mononucleated 12c cells, may give rise to a hexanucleated or a mononucleated 24c cell, respectively; both these types of cells were detected by combining microfluorometric and microscopic analyses. Small micronuclei, predominantly observed in hexanucleated cells, can contribute to the imbalance of the DNA content of the individual nuclei. Mononucleated 12c cell origin would be attributable to bipolar mitotic processes, in the absence of cytokinesis, occurring in 6c cells (12c after the S phase), in which all chromosomes would be segregated in a single telophasic nucleus. Subsequently, these 12c cells appeared to be capable of undergoing mitotic division, as demonstrated by the presence in NIHs cultures of 12c nuclei linked by interchromatin bridges after cytokinesis. Hexanucleated 12c cells, with balanced 2c DNA content in each nucleus, were not detected. This absence could be caused by the inability of trinucleated 6c cells with aligned nuclei (derived from the fusion of three 2c cells) to duplicate their DNA and enter mitosis. This could be a possible consequence of the inability to synchronize their three nuclei, which may be in different stages of the cell cycle. After PEG treatment of NIHs cultures, in addition to 10-12c and 24c cells, the 16c subpopulation also increased in percentage compared to the control. Cells with 16c DNA content were detected as binucleated and tetranucleated, with two 8c nuclei or four 4c nuclei, respectively. As an alternative to possible fusion events involving two 8c or four 4c elements, the origin of the two 16c cell types could be attributed to mitotic processes occurring in mononucleated 8c cells (16c after the S phase) or in binucleated 8c cells (with two 4c nuclei; 16c after the S phase), respectively, detected in NIHs culture. In both cases, the mitotic events would not be accompanied by cytokinesis. Regarding the origin of binucleated 4c cells (with two 2c nuclei), in addition to the fusion of two 2c cells, the occurrence of mitotic events in a 2c cell (4c after S phase), without cytoplasm division, can be considered. This would lead to the genesis of a cell with two 2c nuclei. As an alternative, a single 4c nucleus could reform in telophase. Phenomena of this kind could also be considered to interpret the presence of mononucleated 8c elements: in this case, the nuclei involved would have 4c and not 2c DNA content.

Some studies carried out in recent decades have converged to indicate that among the different checkpoints defined in cell cycle progression, the ‘‘checkpoint of tetraploidy’’ is also included [35]. Its presence would have the meaning of avoiding the proliferation of 4c cells and the genetic instability that could result from it. This could lead to the opinion that cells with tetraploid DNA content have poor proliferative capacity. On this topic, the results obtained in some works do not always appear in agreement with each other. In some cases, experiments on different cell models indicated that, especially in transformed cells, tetraploid binucleated cells (with two 2c nuclei after cytokinesis failure) retain their proliferative capacity and can produce multinucleated cells [36]. This evidence would be in agreement with the aforementioned hypothesis in which we considered that 4c NIHs cells would be progenitors of 8c and 16c cells.

The proliferating and nonproliferating cell subpopulations constituting the NIHs culture were found to be differently sensitive to the action of the cytostatic agent vinblastine. Compared to the conditions in the absence of VBL, in both PEG-untreated and PEG-treated cultures, the accumulation of 4c cells induced by the drug can be the consequence of a mitotic block in the 2c cells (4c after the S phase). On the other hand, the lack of accumulation and the decrease in 8c and 16c subpopulations induced by VBL could be partly linked to the lower proliferative capacity of 4c (8c after the S phase) and 8c (16c after the S phase) cells compared to that of 2c cells. Furthermore, variable cell death mechanisms may play a role in the dynamics of these subpopulations. It can be emphasized that in NIHs culture, only cells with DNA contents near 2c, 4c and 8c show, albeit at a low percentage, the features of conventional apoptotic death (type I PCD); a higher value of apoptosis was detected in the 8c subpopulation. Moreover, fluorescence microscopy observations performed during DNA microfluorometric analysis, in both PEG-untreated and PEG-treated NIHs cultures, allowed us to also detect that in 4c and 8c cells (after the S phase in 2 c and 4 c cells, respectively) and in 12c (after the S phase in 6c cells) more limitedly, a fraction of them showed the presence of chromosomal structures dispersed in the cytoplasm, mainly derived from altered metaphases, after VBL action on mitotic spindle. For these cell subpopulations, the occurrence of apoptotic death events or, alternately, alterations in mitotic processes may be dependent on the phase of progression in the cell cycle. The appearance of chromosome aggregates could be followed by cell death mechanisms at least partly related to mitotic catastrophe [33]. On the other hand, the disappearance of 12 c and 24 c cells after VBL incubation of the PEG-untreated cultures could be due to the particular drug sensitivity of the microtubular network and mitotic apparatus of the proliferating 6 c and 12 c cell fractions. The limited number of 12 c aggregates of chromosomal structures induced by VBL in PEG-treated cultures compared to their absence in PEG-untreated cultures could be related to the higher incidence of 6c cells (12 c after S phase) after PEG-induced cell fusion.

The absence or presence of 12 c cells, which showed some proliferative activity, could also affect the absence or presence of the 24 c subpopulation. Indeed, in NIHs cultures previously treated with PEG, in consideration of the higher percentage incidence of 6c and 12c cells, VBL incubation resulted in a decrease in 12 c cells, accompanied by a decrease in 24c cells.

Compared to the VBL absence condition, the subpopulation of 16c cells tended to decrease in percentage after drug action in both PEG-untreated and PEG-treated NIHs cultures. Following drug incubation, the percentage incidence of these cells can be influenced by the limited numerical contribution given by the proliferation of 8 c cells (eventually, they become 16 c after the S phase). After VBL action, in both PEG-untreated and PEG-treated NIHs cultures, fluorescence microscopy observations performed during DNA microfluorometric analysis allowed us to detect a very limited number of aggregates of chromosomal structures near the 16 c value. For this DNA content, it was possible to more frequently detect particular nuclear situations with the appearance of a high number of large micronuclei/nuclear fragments with heterogeneous dimensions per cell. This nuclear condition could be attributed to the probable greater fraction of nonproliferating 16c cells. According to this same interpretation, micronucleations with overall DNA content close to the 24 c value could be representative of a subpopulation consisting of nonproliferating 24 c cells, present in both PEG-untreated and PEG-treated NIHs cultures. In support of this hypothesis, it would also be the lack of an appreciable number of cells with 32 c and 48 c DNA content deriving from the potential proliferation of 16 c and 24 c cells, respectively.

Based on the above considerations, the presence of giant cells with a high level of multinuclearity and DNA content greater than 24 c would be exclusively due to cell fusion events occurring after PEG treatment. In these cultures, VBL incubation resulted in the disappearance of cells with 64 c, 110 c, and 140 c DNA content. Since no specific degenerative mechanisms have been identified in these larger cells, the presence of nuclear fragmentations or large micronuclei would seem to be more typical of less proliferating polyploid cells with intermediate dimensions (within subpopulations with DNA content in the 10c—24 c range). In these cells, the large micronuclei or nuclear fragments could mainly originate from alterations of the interphasic nucleus rather than from mitotic anomalies. In support of this hypothesis, fluorescence and electron microscopy makes it possible to detect introflexions and budding of the nuclear envelope, which could precede the nuclear fragmentation phase, even in the absence of VBL incubation. In some cases, during the progression of this process, detaching nuclear portions appear to be still connected to the main nucleus through thin chromatin bridges. In VBL-incubated NIHs cultures, interphasic nucleus/i instability of cells with intermediate dimensions would be accentuated by the drug action through the involvement of cytoskeletal intermediate filaments that would undergo significant modifications as a consequence of microtubular network depolymerization (discussed later). Alterations in nuclear structure could be part of the modifications that may precede the occurrence of cell death events. In cells with a DNA content ranging from 10 to 24 c, present in both untreated and PEG-treated NIHs cultures, the nuclear anomalies appeared to be predominantly related to some cytoplasmic features (discussed later) typical of autophagic cell death. In PEG-treated cultures, the number of cells with cytoplasmic autophagic features was not significantly increased compared to that in PEG-untreated cultures. Even in the absence of VBL incubation, the overall analysis of degenerative events indicated considerable variability with the involvement of mono- and multinucleated NIHs polyploid cells. In comparison to their diploid counterparts, polyploid cells can show, in general, different types of karyological modifications (e.g., micronuclei of variable size, multilobulated and fragmented nuclei). In some cases, notable dynamic changes, such as a reduction in the number of small micronuclei and an increase in furrowed nuclei with variable shapes, may appear. According to some author opinions, this kind of nuclear alteration may represent a morphological feature related to mitotic catastrophe [33]. In our opinion, the presence of deep nuclear notches may indicate stages of probable transition toward other anomalies and the expression of an intermediate phase characterized by the appearance of nuclear lobes and vesicular budding of the nuclear envelope. At a later stage, heterogeneously sized micronuclei could arise from these protrusions. As reported by other authors, nuclear lobulation and fragmentation are commonly occurring features of various tumor cell lines [37, 38]. In NIHs culture, nuclear fragments or large heterogeneous micronuclei are distinguishable from small micronuclei of probable mitotic origin. In other cell models, lobulation and morphological and structural changes in the nucleus have been related to the perturbation of the cytoskeletal network involving, in particular, the reorganization of the centrosome-microtubule complex [37].

Even in the absence of VBL action, but with an increase after incubation with this drug, the nuclear morphology changes of intermediate polyploid NIHs cells appeared to be concomitant with significant cytoplasm modifications. In these cells, acridine orange vital staining for lysosomal and autophagic activity evaluation showed the presence of bright red cytoplasmic granulations, indicative of autodigestive events.

Furthermore, TEM ultrastructural analysis showed that nuclear changes were associated with remarkable modifications of the cytoskeleton, which also involved the intermediate filament component. Extensive areas of the cytoplasm appeared to be strongly altered with the presence of empty or full vacuoles with heterogeneous content. This situation could be an expression of a kind of hybrid form of degeneration that lies between autophagic cell death and paraptosis [39].

In these two forms of nonapoptotic programmed cell death, the typical internucleosomal fragmentation of DNA produced by the activity of endonucleases activated through the caspase pathway is not described. However, some authors reported a form of PCD in which somato-lactotrope pituitary GH4Cl cells [40, 41] showed morphological characteristics with close similarities with paraptosis, from which it differs in the presence of internucleosomal DNA fragmentation, which is in this case caspase-independent [40, 42].

This emphasizes that certain modalities of cell death can partially overlap but differ for the not identical signaling pathways. This would be consistent with the type of nonapoptotic cell death that we have detected in some NIHs cell subpopulations. In this case, the appearance of dimensionally heterogeneous micronuclei (or nuclear fragments) is associated with DNA fragmentation, as indicated by the positivity of the TUNEL reaction and the presence of intense vacuolation of the cytoplasm.

Moreover, the heterogeneity of vacuole features can be related to their different origins. The electron-dense content (such as residual bodies and myelin figures) inside them could reveal the nature of secondary lysosomes and express an intense autodegradation of organelles and endomembranes. Alternately, the vacuoles that appear to be empty could have an acidic or nonacidic nature and originate from different cellular compartments by different mechanisms: they could arise from the complete digestion of secondary lysosome content or vacuolation can affect the endoplasmic reticulum. The ultrastructural investigation further indicated that vacuoles that appear to be empty can coalesce with each other and form large areas of cytoplasmic discontinuity, which could also be independent of autophagic processes. The presence of vacuolations and autodigestive phenomena, which can mark cytopathological conditions leading to cell death, has been detected with higher frequency in NIHs cells as a consequence of the cytotoxic VBL stimulus. In some cases, tubulin paracrystalline structures can be observed in cytoplasmic areas with strong extensive vacuolation.

Although the mechanism underlying the tight packing of tubulin molecules has not yet been fully elucidated, it can be reasonably hypothesized that the assembly of the paracrystal in a specific area of the cytoplasm subtracts free tubulin from the normal equilibrium of its cytosolic concentration, preventing the formation of normal microtubules. This interference with microtubule polymerization could be reflected in the organization and localization of endomembrane systems within the cytoplasm. Additionally, in nonmitotic cells, it has been previously shown that VBL, as well as other alkaloids, is capable of altering the microtubular network, determining the formation of spheroidal cavities and reducing its mass with changes in cell morphology [43].

Furthermore, these microtubular network alterations could have repercussions on the overall rearrangement of the other components of the cytoskeleton.

In VBL-treated NIHs cells, ultrastructural analysis also showed intermediate filament bundles in the cytoplasm areas, which appeared to be strongly altered with vacuolation coalescence. These ultrastructural aspects, together with the morphocytochemical features detected after acridine orange staining, can indicate, especially in intermediate cells, the presence of active degenerative phenomena that can contribute to a loss of cytoplasmic mass and cell body reduction. This hypothesis would be supported by the drastic morphological changes detected through phase contrast microscopy in some intermediate/large polyploid cells. From this point of view, the images in Fig. 11 may represent, in our opinion, a possible sequence of dynamic events that could characterize the key phases expressed during the execution of a cell death program aimed at dismantling the cell body. According to this proposed interpretation, the cells could proceed on the degenerative pathway by integrating cytoplasm reduction (also through self-digestion processes) with an evident rearrangement of cytoskeleton, leading to the acquisition of nerve cell-like morphology before their detachment from the substrate. Giant mononucleated and multinucleated cells with similar morphological features have been described in previous works, in which HEY and MDA-MB-231 epithelial cancer cell lines were used. In these cultures, giant cells exhibited numerous subtle extensions assuming a neuron-like morphology [44]. As reported by these authors, this particular morphology was displayed by giant cells in relation to asymmetric cell division processes, in which the branches of these cells were involved in budding and bursting patterns that led to the release of different daughter cells. This particular form of cell division, called neosis or depolyploidization by previous investigators, could play a role, also in vivo, in regulating tumor heterogeneity and contribute to the generation of cancer stem-like cells [45, 46]. In our cell model, neuron-like morphology seemed to be predominantly acquired by some intermediate/giant cells in relation to degenerative events. As a possible alternative (or concomitance) to neosis, neuronal-like NIHs cells take on a rounded morphology and tend to detach from the growth substrate after branch retraction. This kind of mechanism could be part of programmed cell death expression in elements with very extensive cytoplasm and strong adhesion to the growth substrate. As reported in the Results section, morphological observations, carried out by phase contrast microscopy during and after culture trypsinization, showed a higher adhesive capability of intermediate/large cells that detach with more difficulty than small cells.

Therefore, in giant cells, drastic remodeling of the cell structure to perform the execution phases of the programmed cell death process would be needed, involving also the lysosomal compartment [47]. In NIHs culture, polyploid giant cells, with DNA content values ranging from 64 to 140c, tend all to be lost in the different experimental conditions; however, among these cells, signs of degeneration were observed only in the 64c subpopulation. Furthermore, according to what was previously described [44], some NIHs cells with neuronal-like morphology belonging to this same subpopulation can show budding phenomena on the thin ramifications of the cytoplasm. These dynamic processes could represent a survival mechanism by which large cells could subdivide, at least in part, their cell body with the genesis of small daughter cells with a low ploidy level (2c-4c DNA content). It cannot be excluded that these processes can also occur in giant cells with DNA content greater than 64c. These cells with a higher number of nuclei present in PEG-treated NIHs cultures disappeared after VBL incubation, despite showing a very organized cytoskeleton and a high adhesive capacity related to extensive cytoplasm.

Regarding VBL activity, it is not possible at present to provide conclusive information on several aspects related to the effects on NIHs cells. Beyond VBL specific action on the M phase, the effects on the other phases of the NIHs cell cycle need to be further investigated. Furthermore, the influences on nuclear fusion phenomena and endoreduplication processes that may concern the dynamics of mononucleated polyploid cells need to be clarified. In fact, as alternative to nuclear fusion, cells with a single nucleus can be generated through a mechanism that uncouples DNA duplication from mitotic cell division. As known, endoreduplication can occur through endocycling, in which periods of S and G phases alternate with no cell division. Furthermore, some studies on tumor cells treated with antimitotic drugs, such as colcemid and vinblastine, have demonstrated that endoreduplication can be a cellular process used as a means to survive mitotic catastrophe or genotoxic stress, as an alternative to apoptosis[48, 49].

In the case of NIHs cells, by examining the data collected so far, the polyploid mononucleated cells have shown an oscillating trend in the different cellular subpopulations and in relation to the different experimental conditions. This does not allow us until now to have specific information on a process such as endoreduplication whose data can contribute to clarifying the dynamics of appearance and disappearance of polyploid cells in the culture of NIHs cells.

Regardless of the mononuclear conditions and using other cellular models, in some works on the mechanisms of drug resistance induced by the increase in cell ploidy values of some tumors (whose DNA content value was not indicated), it has been shown that although most giant polyploid cells undergo death events, alternately, a fraction of them can contribute to the negative evolution of the tumor, being able to produce a progeny of viable cells. Then, in this case, the number decrease of giant elements may be partly due to the subdivision of their large cell body into cells with a lower ploidy level, which include some proliferating cells near diploidy [19, 44, 45].

Regarding the dynamics related to the fate of NIHs polyploid cells with particular reference to degenerative and depolyploidization events, conclusive elements could be provided by the use of real-time microscopy and time-lapse imaging techniques.

## Conclusions

While highlighting the limitations of in vitro studies in reproducing the complexity of tissue and organ systems, PEG-treated NIHs cultures can represent a model of heterogeneous subpopulations originating from cell fusion and division process anomalies, which could be used in screening programs for molecules with different pharmacological activities. As a consequence of different cell features related to the different DNA content, NIHs subpopulations can provide variable responses to stimuli internal to the culture or induced by exogenous pharmacological treatments able to induce cell degeneration and death. Canonic, autophagic-like, or hybrid forms of cell death can also depend on the ability of the cells to progress through the cell cycle and may influence the persistence and fate of polyploid descendants, which is also related to chemotherapeutic agent action. The different cell subpopulations constituting the NIHs culture, on the basis of their state, can use different ways of expression to activate self-destruction. Conventional apoptosis seems to be a mechanism involved, especially in the removal of cells with higher proliferative activity and a reduced dimension, while autophagic-like or alternative degenerative phenomena appeared to be involved in the removal of polyploid cells with intermediate and larger sizes. Concerning the possibility that the different cell features can modulate the expression of alternative or hybrid forms of cell death, it can be underlined that autophagic events could alter conventional apoptosis progression, making one or the other form of cell death prevail. This highlights the need to clarify the role of autophagy in the regulation of processes related to the cell degeneration machinery and the interconnection with apoptosis or other forms of cell death. In the larger giant NIHs cells, with a DNA content greater than 64c, no aspects related to cell death events were clearly detected under the different experimental conditions. This could support the hypothesis that the decrease and disappearance of these subpopulations would be partly related to possible cell body fragmentation as an alternative to the activation of a cell death program, with the genesis of a progeny of smaller cells with a lower ploidy level. The possibility of clarifying these aspects and the expansion of knowledge in this area could provide the basis for the development of new therapeutic strategies in the treatment of tumors characterized by marked cellular heterogeneity.

## Methods

### Cell cultures

The NIHs cells were cultured in Dulbecco’s modified Eagle’s medium (Sigma-Aldrich) supplemented with 10% fetal calf serum (Gibco, Life Technologies), 2 mM L-glutamine (Gibco, Life Technologies), 100 units/ml penicillin (Gibco, Life Technologies), and 100 μg/ml streptomycin (Gibco, Life Technologies) at 37 °C in a humidified atmosphere containing 5% CO_2_. The NIHs culture used in this work was previously obtained after prolonged (7–10 days) serum deprivation (culture medium containing 0.5% fetal calf serum) from the NIH3T3 mouse embryo fibroblast line. The withdrawal of serum caused massive cell detachment and death of almost all cells constituting the culture. A small fraction of surviving cells, when serum was restored, re-entered the cell cycle and, after recovery of the normal proliferative activity, showed a higher presence of polyploid/aneuploid cells [20] with respect to the original NIH/3T3 cell line.

The cultures were propagated by trypsinization by incubating the cells at 37 °C for times ranging from 7 to 15 min. The first trypsinization was performed 30 h after PEG treatment.

Trypsin (Gibco, Life Technologies) was typically used at a concentration of 0.25% in calcium- and magnesium-free Dulbecco’s phosphate buffered saline (PBS) (Gibco, Life Technologies). The cultures were analyzed as a subconfluent monolayer, both in control and after treatment conditions.

### PEG-induced cell fusion

The ability of PEG to fuse biological membranes is primarily referred to as its chemical nature as a polymer capable of achieving a high level of hydration and therefore functioning as a dehydrating agent. Referring to this property and the consequent subtraction of water from cell surfaces, PEG can put plasma membranes close to tightening molecular contact. Then, to induce fusion, it is necessary that lipid monolayer packaging is disorganized with the mixture of lipids in the bilayers of touching membranes [50].

In this work, PEG-mediated cell fusion was performed in a single-step procedure: NIHs cells were incubated with 37% PEG 6000 MW (Merck-Schuchardt, Hohenbrunn, Germany) in Dulbecco’s modified Eagle’s medium, pH 8.2, for an exposure time of 10 min at 37 °C in a humidified atmosphere containing 5% CO_2_.

The best experimental conditions for cell fusion, which guaranteed an increase in polyploid/aneuploid multinucleated cells and the maintenance of acceptable cell viability conditions (cell viability was as high as 88,2 ± 3.2%), were determined based on preliminary experiments that allowed us to define treatment modalities in terms of both PEG concentration and incubation time. For PEG-mediated fusion, NIHs cells were grown in T-25 cm^2^ culture flasks up to the achievement of a confluence of approximately 75%, and the most favorable incubation times ranged from 10 to 15 min, without showing significant differences in terms of fusion efficiency and cell viability in this range. Subconfluence permitted the maintenance of some proliferative capability of the culture and, at the same time, rented the cells (especially in some areas of the growth substrate) near enough to allow contact for PEG-induced fusion. After treatment, the PEG solution was removed, and the cells were washed twice in PBS and maintained in complete medium for 30 h. This time was also chosen to obtain information on the cytokinetic aspects of the new cell subpopulations induced by the fusogenic agent. After PEG treatment, cell fusion products were evaluated by phase contrast and fluorescence microscopy. To determine the percentage incidence of polyploid cells, a mean number of 500 cells were counted; cell viability was determined by trypan blue exclusion. The data obtained by morphological analysis and fluorometric evaluations 30 h after PEG treatment represented the reference situation (control). After this time, some cultures were subjected to trypsinization for propagation, while other cultures were incubated with vinblastine (VBL).

### Vinblastine incubation

Cell cultures were incubated with 2 μg/ml VBL (Sigma-Aldrich) in complete medium for 24 h. The drug was added to both untreated (control) and PEG-treated subconfluent cultures.

### Microscopy and photographic documentation

Photographic documentation was obtained using a Zeiss Axiovert 40C inverted phase contrast microscope (Carl Zeiss Jena GmbH, Jena, Germany) in a conventional way and suitable lighting to obtain an interference contrast effect. A Nikon Eclipse 600 microscope (Nikon, Kanagawa, Japan) equipped for epifluorescence observation of fluorochromized samples was used.

### Evaluation of polyploid cells by microfluorometry of DNA content

NIHs cells grown under different experimental conditions on coverslips were fixed with 70% ethanol for 30 min at 4 °C. Nuclear DNA content was determined using Hoechst 33,342 (Sigma-Aldrich) or acridine orange (AO) (Sigma-Aldrich) fluorochromes.

For DNA staining with Hoechst 33342, the cells were incubated with a fluorochrome solution [2 µg/ml in PBS (pH 7.4)] for 30 min at room temperature. For the AO staining procedure, see below.

The amount of DNA per cell was analyzed by measuring the fluorescence emitted by every single nucleus. For this analysis, a Nikon P II cytoflurometer (Nikon, Kanagawa, Japan) was employed using a 100 × oil-immersion objective.

In the microfluorometric apparatus, an appropriate system of diaphragms permitted the selection of single nuclei, and the fluorescence was quantitatively analyzed after automatic subtraction of the background brightness. The operations of alignment and focusing of the nuclei were preliminarily conducted under phase contrast in low light conditions to avoid photo decay.

In a series of parallel samples, the cells were fluorochromized with acridine orange to better correlate the DNA content to cell morphology and different nuclear conditions (multinucleation and anomalies). In this way, images related to a specific cellular situation were acquired after the DNA content microfluorometric measurements.

For each experimental condition, three slides were examined, and 500 cells per slide were measured. The DNA content results were represented as fluorescence intensity histograms.

A histogram of the DNA content of NIH-3T3 culture before serum starvation was inserted in the results. During this microfluorimetric measurement, a suspension of lymphocytes separated by mouse blood [49, 51] was used as an external reference standard to derive the position of the 2c value in abscissa. Lymphocytes cytocentrifuged (200 × g for 10 min) on microscope slides previously coated with Poly-L-Lysine Hydrobromide (SIGMA, St. Louis, MO, USA) (300 μg/ml in PBS) were simultaneously measured under the same instrumental conditions used for NIH-3T3 cells.

The results of the measurements of the DNA content of the two samples were reported in the same graph, and the value of their ratio calculated based on peak channel numbers allowed us to detect that the NIH/3T3 cells have, in the G0/G1 phase, DNA content in the range 3c-4c. The cells of this line are therefore hypertriploids. In the case of the NIHs line, given the presence of different cellular subpopulations and to simplify data representation, cells in G0/G1 were arbitrarily considered diploids, with the first peak in the logarithmic scale of the cytofluorimetric histogram indicated as 2c. A similar criterion of representation of cellular ploidy data for NIH-3T3 cells has been previously used by other authors [52].

### Acridine orange staining

#### DNA microfluorometric measurements

Acridine orange is a metachromatic fluorophore useful for nucleic acid analysis. Through the application of standard protocols (see below), the use of this fluorophore offers simultaneous cell staining of DNA and RNA. In this way, it is possible to distinguish and measure separately the fluorescence of the two nucleic acids due to AO intercalation in the DNA double helix or electrostatic interaction with single-stranded RNA and stacking of dye monomers. Therefore, the cytoplasm shows a dark red (> 630 nm) fluorescence of RNA, while in the nucleus, acridine orange fluoresces bright green (525 nm) after interaction with DNA and dark red after interaction with RNA of nucleoli.

NIHs cells were stained as follows, using an adjustment of the method of Darzynkiewicz [53] applied to cells grown on coverslips in multiwell plates. After fixation with 70% ethanol for 30 min at 4 °C, cells were washed in buffer A (0.1% Triton X-100, 0.2 M sucrose, 10^–4^ M EDTA, and 2X 10^–2^ M citrate phosphate buffer, at pH 3.0) (Sigma-Aldrich). Thereafter, 1 ml of buffer A was added to each well for 1 min. For staining, 2 ml of the solution containing 50 µg/ml acridine orange, 0.1 M NaCl, and 10^–2^ M citrate–phosphate buffer (pH 3.8) were added to the cells. After 15 min of incubation at room temperature, single-cell fluorescence intensities were measured with a Nikon P II cytoflurometer. To measure nuclear fluorescence emission, red fluorescence of the cytoplasm was excluded through a variable diaphragm between the specimen plane and the ocular, limiting the measurement of the fluorescent image to the nucleus only.

#### Autophagy detection

As an alternative to the previous procedure, AO can be used in vital stains as a marker of autophagy. Under specific incubation conditions (low concentration and neutral pH), this fluorochrome accumulates in the acidic compartments of the cells in a pH-dependent manner. Within acidic vesicles (assuming a pH value of 4.5–5.0) such as lysosomes and autolysosomes, the molecule is protonated, and within the organelle, it forms aggregates that emit bright red fluorescence. This metachromatic shift is due to the dimerization of AO at high concentrations inside acidic vesicles [54]. In the nucleus, acridine orange fluoresces weakly green. This is a common method used to visualize acidic intracellular compartments, quantify acidic vesicular organelles, and evaluate their distribution in relation to events such as autophagy [55].

For the staining procedure, cells were incubated for 10 min at 37 °C with AO used at a concentration of 2.5 µg/ml in culture medium (pH 7.4). The samples were examined after washing in PBS to remove excess dye. Under these incubation conditions, the membrane permeability to protonated AO is very low, and therefore, it is possible to clearly distinguish brilliant red fluorescent granulations in the cytoplasm. This assay can be an alternative or complementary to other methods for determining acidic vesicles that increase during autophagy induction. Other works indicated that red fluorescent cytoplasm granulations obtained after AO staining, under the conditions described above, correlated with the distribution of fluorescent-tagged LC3 [55].

In the present work, autophagy was quantified based on the mean number of cells displaying intense punctuate red staining of the cytoplasm. For each experimental condition, at least 500 cells from three different slides were evaluated. The analysis was performed under a fluorescence microscope using a 50 × oil-immersion objective.

#### Apoptosis detection

The morphological detection of the different forms of PCD was carried out during the measurements of the DNA content after acridine orange or Hoechst 33342 fluorochromization, according to the procedures already reported above. Regarding type I PCD, nuclei showing morphological alterations, including chromatin condensation and its margination against the nuclear envelope, nuclear shrinkage, and karyorrhexis, were considered apoptotic. Nuclei with these characteristics were expressed as a percentage of the total cells evaluated. The occurrence of DNA fragmentation was assessed through the TUNEL (terminal deoxynucleotidyl transferase dUTP nick end labeling) assay. Briefly, NIHs cells grown on glass coverslips were fixed with 4% (w/v) paraformaldehyde (Sigma‒Aldrich) and analyzed using a commercial kit (TUNEL In Situ Cell Detection Kit, AAT Bioquest) according to the manufacturer's protocol (#22,844, AAT Bioquest, Inc. CA, USA). Samples were incubated with 50 μl of TUNEL working solution (0.5 µL of 100X Tunnelyte^™^ Red was added to 50 µL of reaction buffer) for 1 h at 37 °C in a dark chamber. After removing the TUNEL working solution, the cells were washed with PBS, and to establish the cell fraction positive for the reaction, nuclei were counterstained with Hoechst 33342 (ex/em: 355 nm/465 nm). The TUNEL red fluorescence signal was analyzed using a fluorescence microscope with a TRITC filter set (ex/em: 540 nm/590 nm).

#### Cytoskeleton network labeling

Some details of the cytoskeleton related to cell adhesion were acquired through fluorescence microscopy after the application of immunofluorescence techniques.

#### Microtubule network

NIHs cells grown on glass coverslips were fixed in 3% paraformaldehyde (Sigma-Aldrich) in PBS containing 1 mM EGTA and 1 mM MgCl_2_ (Buffer A) for 30 min at room temperature. After fixation and washing in Buffer A, the cells were permeabilized with Triton X-100 (0.2% in Buffer A) for 15 min at room temperature. Cells were incubated with anti-tubulin antibodies (1:2000, Sigma #T5168) for 1 h in a humid chamber. After washing in Buffer A, the cells were incubated with goat anti-mouse Alexa 488 antibodies (1/800, Molecular probes # A-11001) for 1 h in a humid chamber. In indirect immunofluorescence techniques, negative reaction controls were obtained by omitting the incubation in the presence of the primary antibody. In all fluorescence microscopy specimens, nuclear counterstaining was obtained with Hoechst 33342 (2 μg/ml in PBS) for 10 min at room temperature. After washing in Buffer A, coverslips were mounted in a 1:10 (v/v) mixture of PBS/glycerol containing *p*-phenylenediamine (Sigma-Aldrich) as an anti-fading agent. For fluorescence microscopy, the following conditions were used: 490 nm/525 nm (excitation/emission) for Alexa 488 and 355 nm/465 nm (excitation/emission) for Hoechst 33342.

#### Microfilament network

NIHs cells grown on glass coverslips were fixed in 3% paraformaldehyde in PBS containing 1 mM CaCl_2_ and 1 mM MgCl_2_ (Buffer A) for 30 min at room temperature. After fixation and washing in Buffer A, the cells were permeabilized with Triton X-100 (0.2% in Buffer A) for 15 min at room temperature. Actin microfilament visualization was performed by incubating the cells in the presence of TRITC-conjugated phalloidin (dilution 1:50) (Sigma-Aldrich) for 45 min in a humid chamber.

Nuclear counterstaining was obtained with Hoechst 33342 (2 μg/ml in PBS) for 10 min at room temperature. After washing in Buffer A, coverslips were mounted in a 1:10 (v/v) mixture of PBS/glycerol containing p-phenylenediamine as an anti-fading agent. For fluorescence microscopy, the following conditions were used: 540 nm/590 nm (excitation/emission) for TRITC; 355 nm/465 nm (excitation/emission) for Hoechst 33342.

All fluorescence micrographs were obtained with a digital camera (Olympus ColorView III u) (Tokyo, Japan) connected to an epifluorescence microscope (Nikon Eclipse 600*)* equipped with a 100 Watt mercury lamp.

#### Transmission electron microscopy

NIHs cells grown in 75 cm^2^ culture flasks under various experimental conditions were washed in phosphate-buffered saline and then fixed for 2 h with 2.5% glutaraldehyde (Sigma-Aldrich) in 0.1 M sodium cacodylate buffer (pH 7.4) (Sigma-Aldrich) at 4 °C. The fixed samples were scraped off with a spatula, transferred to sterile tubes and centrifuged at 300 × g for 10 min.

The pellets were washed in the same buffer containing 5% sucrose and postfixed for 90 min with 1% osmium tetroxide (Sigma-Aldrich) and 1.6% potassium ferrocyanide (Sigma-Aldrich) in sodium cacodylate buffer (pH 7.4) at 4 °C. The cells were then resuspended in 1 ml 2% agarose and centrifuged at 300 ×*g* for 10 min at 35 °C. After 1 h at 4 °C, the samples were fragmented, dehydrated in an increasing ethanol series, and embedded in epoxy resin (Agar 100) (Agar Scientific, Stansted Essex, UK) at 60 °C for 48 h. Ultrathin sections were mounted on 200 mesh copper, double stained with uranyl acetate and lead citrate and examined under a Zeiss EM 900 transmission electron microscope operating at 80 kV (Carl Zeiss Jena GmbH, Jena, Germany). The basic ultrastructural analyses focused on nuclear morphology and some subcellular cytoplasmic aspects indicative of cell degeneration.

### Statistical analysis

Statistical analysis was performed using SigmaPlot Software (Systat Software Inc., San Jose, CA). The measurement data were expressed as the mean ± standard error (SEM). A t test was used for comparisons between two groups. P < 0.05 was considered statistically significant.

## Data Availability

All data generated or analyzed during this study are included in this published article.

## Abbreviations

PEG: Polyethylene glycol
VBL: Vinblastine sulfate
AO: Acridine orange
TEM: Transmission electron microscopy
